# Non-B DNA structures and their contributions to genetic diversity, aging, and disease

**DOI:** 10.1093/nar/gkag084

**Published:** 2026-02-10

**Authors:** Eleftherios Bochalis, Irene Dereki, Guliang Wang, Argyro Sgourou, Karen M Vasquez, Ilias Georgakopoulos-Soares

**Affiliations:** Division of Pharmacology and Toxicology, College of Pharmacy, The University of Texas at Austin, Dell Paediatric Research Institute, Austin, TX 78712, United States; Biology Laboratory, School of Science and Technology, Hellenic Open University, Patras 26335, Greece; Division of Pharmacology and Toxicology, College of Pharmacy, The University of Texas at Austin, Dell Paediatric Research Institute, Austin, TX 78712, United States; Biology Laboratory, School of Science and Technology, Hellenic Open University, Patras 26335, Greece; Division of Pharmacology and Toxicology, College of Pharmacy, The University of Texas at Austin, Dell Paediatric Research Institute, Austin, TX 78712, United States; Division of Pharmacology and Toxicology, College of Pharmacy, The University of Texas at Austin, Dell Paediatric Research Institute, Austin, TX 78712, United States

## Abstract

DNA is most often found in its canonical B-form double-helical structure, but can also adopt alternative conformations, known as non-B DNA structures. Numerous non-B structures have been characterized, including G-quadruplexes, i-motifs, Z-DNA, hairpins, cruciforms, slipped structures, R-loops, and H-DNA. Non-B DNA motifs are enriched in functional regions, including near transcription start and end sites, topologically associated domains, and replication origins, suggesting their importance in gene regulation, genome organization, and replication. However, these structures are intrinsically prone to error-generating processing, leading to genomic instability and hence have been implicated in the development of human diseases. Here, we discuss recent advances in understanding the biological roles of non-B DNA structures and their contribution to genomic instability in somatic and germline contexts. We highlight how they promote replication stress, transcription stalling, and DNA breaks, resulting in the formation of mutational hotspots. Emerging technologies have enabled the detailed mapping of previously challenging repetitive regions that harbor potential non-B DNA-forming sequences, and are poised to unravel additional contributions in human disease and evolution. Furthermore, we explore the dual role of non-B DNA as a driver of genetic variation that facilitates evolutionary adaptation and as a source of mutations that contribute to tissue dysfunction and aging.

## Graphical Abstract

**Graphical Abstract:**
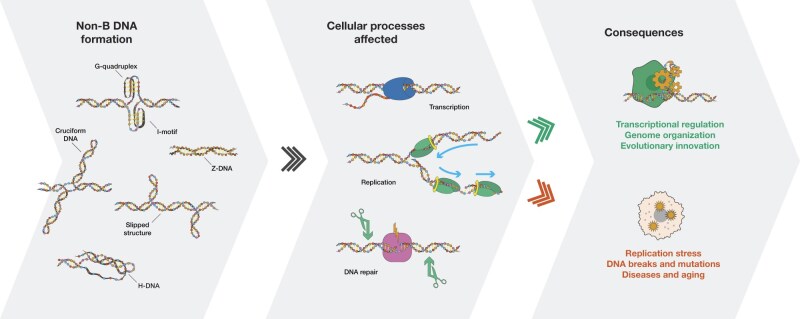

## Introduction

**Figure 1.**
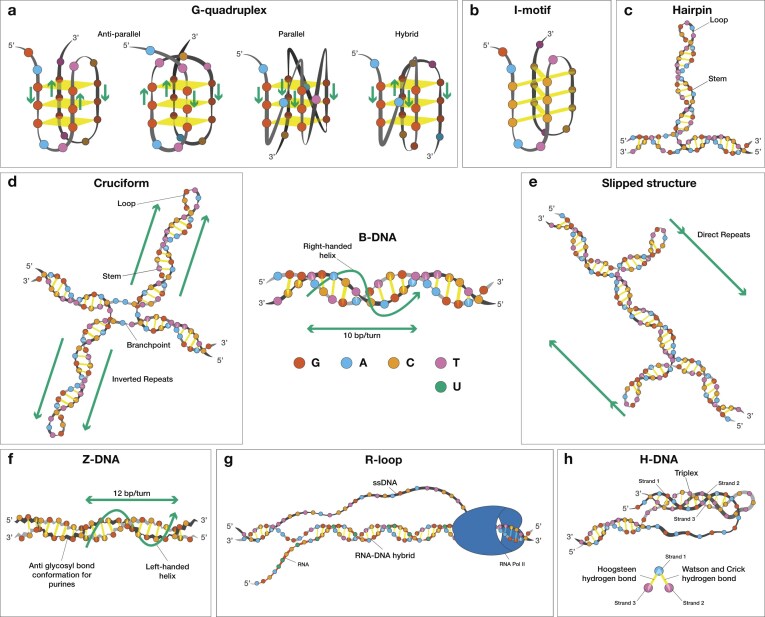
Schematic of non-B DNA structures. Schematic illustrations of noncanonical DNA conformations, beyond the physiological B-DNA form. (**A**) G-quadruplex structure in which stacked guanine tetrads are held together with Hoogsteen hydrogen bonds. (**B**) I-motif structure. (**C**) Hairpin, and (**D**) cruciform formation at inverted repeats. (**E**) Slipped structure formation at tandem repeats. (**F**) Z-DNA structure. (**G**) R-loop formation during transcription. (**H**) H-DNA formation at mirror repeats stabilized by Hoogsteen hydrogen bonds. A Hoogsteen base pair is a type of non-Watson–Crick bond, where the purine base rotates 180° with respect to the helix axis and adopts a syn conformation. B-DNA is depicted at the center of the figure, showing the right-handed structure with 10 bps per turn.

The canonical conformation of the DNA molecule, initially described by Watson, Crick, Wilkins, and Franklin in 1953 [1], is a right-handed double helix with a diameter of ∼2 nm (Fig. 1). Under typical cellular conditions, this is the predominant and most thermodynamically stable structure, commonly termed B-DNA. However, specific sequence motifs, ionic environments, and topological stresses can result in the formation of noncanonical or alternative structures (i.e., non-B DNA) [2–4]. Non-B DNA structures have been implicated in gene regulation, genome organization, recombination, replication, and responses to environmental stresses, among other biological processes [5–11]. Loci that are predisposed to non-B DNA formation include repetitive sequences capable of adopting G-quadruplex DNA, Z-DNA, hairpin/cruciform inverted repeats, slipped DNA structures formed at direct and tandem repeats, and H-DNA mirror repeats (Fig. 1). Non-B DNA structures require negative supercoiling to form; hence, processes such as replication, transcription, and DNA repair promote their formation [12–14]. These motifs are enriched in open chromatin regions, promoters, 5′ and 3′ untranslated regions, near transcription start and end sites, among other regions, and are often associated with transcriptionally active loci [5, 9, 11, 15, 16].

Despite their important regulatory functions, these same non-B DNA structures present a double-edged sword for genomic stability. Mutations in the human genome are not distributed homogeneously [17–20]. Non-B DNA-forming sequences can serve as endogenous mutational hotspots [19, 21], due to their intrinsic genomic instability, and can lead to functional consequences. Such non-B DNA-induced instability has been shown across prokaryotic and eukaryotic organisms [22–27] and has been associated with disease development, including cancer and neurodegenerative disorders in humans [19, 27–34].

Beyond their role in disease, recent evidence suggests that non-B DNA-induced mutagenesis can also affect the process of aging. In somatic cells, *in vivo* models show that non-B DNA structures act as a source of local instability that can increase with age in a tissue-specific manner [35–37]. Such age-associated increases in mutational burden and/or apoptosis can disrupt tissue homeostasis and drive cellular dysfunction, ultimately contributing to the aging process [38, 39]. In germline cells, the same propensity for genetic instability can serve as a template for genetic variation, thereby facilitating evolutionary adaptation. This dual nature underscores the importance of elucidating the functional roles of non-B DNA, both to advance our understanding of the mechanisms of aging and complex diseases, and to enable more precise modeling of evolution [8, 40, 41].

In this perspective, we examine recent advances in understanding the roles of non-B DNA motifs in both genome function and dysfunction. We highlight how recent developments in sequencing technologies can facilitate the examination of non-B-DNA-forming sequences in low-complexity and highly repetitive genomic regions. These previously challenging loci open new avenues for understanding the roles of non-B DNA in disease and evolution. Finally, we examine recent results on the association of non-B DNA with somatic mutations in aging, cancer, and neurodegenerative disorders, and propose directions for leveraging non-B DNA research in therapeutic and diagnostic applications.

## Non-B DNA categories and their detection

Specific sequence features, including guanine-rich tracks, alternating purine-pyrimidine runs, mirror, inverted, and tandem repeats, predispose DNA to adopt non-B conformations [6]. **G-quadruplexes** are nucleic acid structures stabilized by Hoogsteen hydrogen bonds between guanines that form stacked G-tetrads [42, 43] (Fig. 1A). Their cytosine-rich complementary sequence can fold into intercalated motifs (**i-motifs**) (Fig. 1B). However, G-quadruplexes and i-motifs may not coexist due to steric hindrance; the possibility of their coexistence increases with the offset distance [44]. **DNA hairpins** can form at both perfect and imperfect inverted repeats. These repeats consist of two adjacent sequences, one of which is the reverse complement of the other. In hairpin structures, the complementary arms are held together by hydrogen bonds, while the spacer region remains single-stranded (Fig. 1C). A related structure is the **cruciform**, which resembles the Holliday junction that forms during recombination and is composed of two hairpins and a four-way junction [45, 46] (Fig. 1D). **Slipped structures** form at consecutive repeat sequences, in which one repeat unit misaligns with the second repeat unit on the opposite strand, therefore creating single-stranded DNA (ssDNA) and exposed loops [47] (Fig. 1E). **Z-DNA** is a left-handed double-helical structure typically formed at alternating purine and pyrimidine sequences [48, 49] that contains ∼12 bps per helical turn instead of the canonical 10 bps in B-DNA. The point at which a B-DNA motif transitions to a Z-DNA conformation, and vice versa, is termed a B-Z junction, which contains unpaired bases. (Fig. 1F). **R-loops** are three-stranded nucleic acid structures formed when an RNA molecule hybridizes with one strand of DNA, leaving the complementary DNA strand unpaired [50]. They can occur naturally during transcription and play roles in gene regulation; however, excessive R-loop formation can lead to genomic instability and has been associated with several human diseases [51, 52] (Fig. 1G). Another non-B DNA structure is intramolecular **triple-stranded DNA (H-DNA)**, which can form at homopurine-homopyrimidine sequences that exhibit mirror repeat symmetry [53]. In this structure, one DNA strand folds back into the major groove of the underlying double helix and is stabilized by Hoogsteen or reverse-Hoogsteen hydrogen bonds, forming a triple-helical DNA structure with the complementary strand left unpaired (Fig. 1H). Intermolecular triplexes can also form between DNA molecules, RNA molecules, or between a DNA–RNA hybrid [54]. In each case, Hoogsteen or reverse-Hoogsteen hydrogen bonds stabilize the triplex structure. When the third strand is an independent oligonucleotide, it is termed a triplex-forming oligonucleotide.

**Figure 2.**
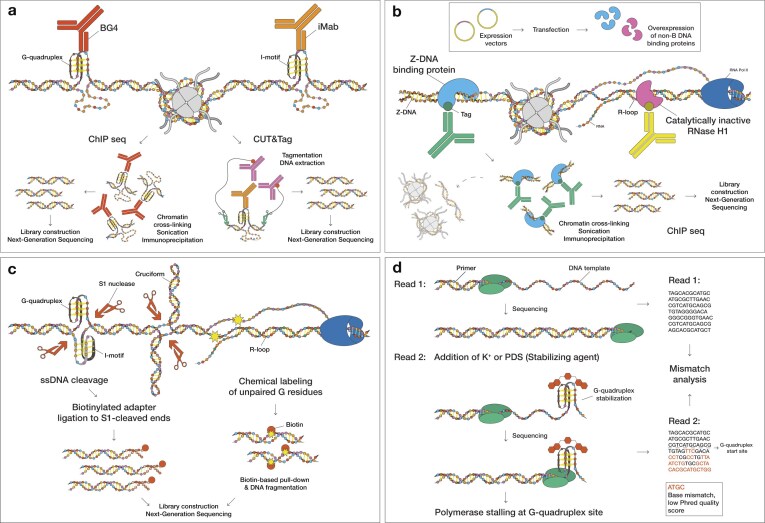
Non-B DNA detection methods. (**A**) Non-B DNA structure-specific antibodies (e.g., BG4 and iMab) enable genome-wide mapping either by immunoprecipitation of antibody-bound, crosslinked chromatin region (ChIP-seq) or by antibody-guided tagmentation at bound sites (CUT&Tag). (**B**) Catalytically inactive or binding-only protein probes with specificity for non-B DNA (e.g., the Z-DNA-binding domain of ZBP1 and catalytically dead RNase H1) linked with an epitope tag are expressed in cells. Non-B DNA motifs are mapped by anti-tag ChIP-seq. (**C**) ssDNA footprinting approaches: in S1-END-seq, the ssDNA-specific nuclease S1 cleaves non-B DNA-associated ssDNA to generate double-strand breaks that are adapter-ligated and sequenced; in KAS-seq, ssDNA is labeled with N3-kethoxal, followed by click-mediated biotinylation, streptavidin pull-down, and sequencing to map R-loop-associated ssDNA. (**D**) In G4-seq, G-quadruplex-stabilizing molecules are used to ensure G-quadruplex formation. Such formation can result in polymerase stalling, thereby reducing sequencing quality.

Various *in vitro* and *in vivo* methods have been developed for the detection of non-B DNA-forming sequences [55, 56]. Some techniques use antibodies [57–59] (e.g., BG4 for G-quadruplexes) [60, 61]; iMab for i-motifs [62, 63] (Fig. 2A), binding proteins (e.g., Z-DNA binding domain of ZBP1 or ADAP1) [64, 65], or inactivated enzymes (i.e., “dead” RNase H for R-loop detection) [66, 67] (Fig. 2B). Also, structure-agnostic techniques exist, which rely on the ssDNA or distorted DNA regions for non-B DNA detection [11, 68, 69] (e.g., S1-END-seq), while others can label ssDNA exposed during transient structural transitions [70] (e.g., KAS-seq) (Fig. 2C). Assays that capture transient ssDNA can provide a high-resolution, genome-wide map of regions prone to adopting non-B DNA conformations [71]. In G4-seq [72, 73], treatment with G-quadruplex-stabilizing or -destabilizing ions/small molecules has been shown to affect the *in vitro* folding potential (Fig. 2D). However, these methods have limitations. Antibody- and ligand-based approaches exhibit binding biases and can sometimes stabilize or destabilize non-B DNA structures [54, 60, 74–76]. Certain antibodies are biased toward sequence recognition and secondary structure identification [77–79]. Run *et al.* (2025) recently summarized different methods used for detecting Z-DNA and discussed their strengths and limitations [80].

Direct detection of non-B DNA structures in the genomes of living cells is even more challenging because they are dynamic and transient. Nevertheless, certain newer approaches have been developed to study this dynamic nature. For R-loops, a genetically encoded sensor consisting of three RNase H1 domains linked to a fluorescent protein can be used for live-cell imaging of cellular R-loops and for the kinetic analysis of R-loop formation [81]. For G-quadruplexes, Galli *et al.* (2022) have developed a fluorophore-linked nanobody that can be expressed intracellularly and detect G-quadruplexes in living cells [82]; Di Antonio *et al.* (2020) have engineered a fluorescent probe that, at low concentrations, enables single-molecule and real-time detection of individual G-quadruplex structures [83]; and Summers *et al.* (2021) have developed a fluorescence lifetime imaging microscopy assay to study G-quadruplex formation in real time [84]. Finally, in-cell nuclear magnetic resonance (NMR) has been employed to study the equilibrium between i-motifs and B-DNA during replication in living cells [85]. Again, most of the techniques mentioned above provide either indirect measurements or require intermediate steps (e.g. cell fixation), which can stabilize or destabilize existing non-B DNA structures [6]. Therefore, it is important to consider that the non-B DNA conformations detected by these experimental methods reflect only the specific conditions under which they were tested.

Experimental techniques have enabled the development of computational algorithms to detect G-quadruplexes [86–95], Z-DNA [96–99], R-loops [100, 101], H-DNA [102–104], and i-motifs [105, 106]. They use sequence motifs, thermodynamic parameters, and machine learning techniques to systematically scan genomes for loci whose sequence composition and predicted thermodynamic stability resemble those of experimentally validated sequences. The experimental data informing these predictions consist primarily of *in vitro* biophysical measurements on purified oligonucleotides and genome-wide non-B DNA maps. These tools can analyze regions inaccessible to experimental methods and enable systematic genome-wide predictions. Beyond stand-alone algorithms, many databases have been created that compile experimentally validated and computationally predicted non-B DNA-forming sequences, offering a valuable resource for integrative analyses across genomes [100, 107–115]. Despite their utility, they have certain drawbacks. Most tools cannot handle cellular contexts (salt concentrations, protein interactions, and chromatin state), whereas different algorithms designed for the same non-B DNA structure can yield inconsistent predictions for the same sequence due to differences in parameters utilized. They typically provide static predictions and cannot capture the transient, dynamic nature of non-B DNA formation; moreover, machine learning approaches may inherit biases from the experimental data used for training.

Note: While these tools can handle high-throughput data at scale and at low cost, it is crucial to keep in mind that they only predict a sequence’s potential to form a non-B DNA structure, and hence experimental validation is required to verify this prediction. When interpreting experimental findings, it is essential to point out that terms such as non-B DNA structures and non-B DNA-forming sequences are often used interchangeably. Careful evaluation is advised to determine whether studies provide evidence for DNA structure formation or for sequences with the potential to form such structures. Evidence for structure formation should also be considered in the context of the experimental design used. Factors such as ionic strength and superhelical tension can vary significantly between in vitro and in cell-based assays. Additionally, non-B DNA structures are highly dynamic; a noncanonical structure may form in one cellular state (e.g., during replication) but be absent in another, depending on factors such as local negative supercoiling and chromatin accessibility.

## Non-B DNA at regulatory hotspots drives genome function

Non-B DNA-forming sequences are inhomogeneously distributed across the human genome [19]. While tandem repeats are primarily found in highly repetitive regions of the genome, most non-B DNA motifs are preferentially positioned and enriched at *cis*-regulatory elements, telomeres, centromeres, and introns.

**Figure 3.**
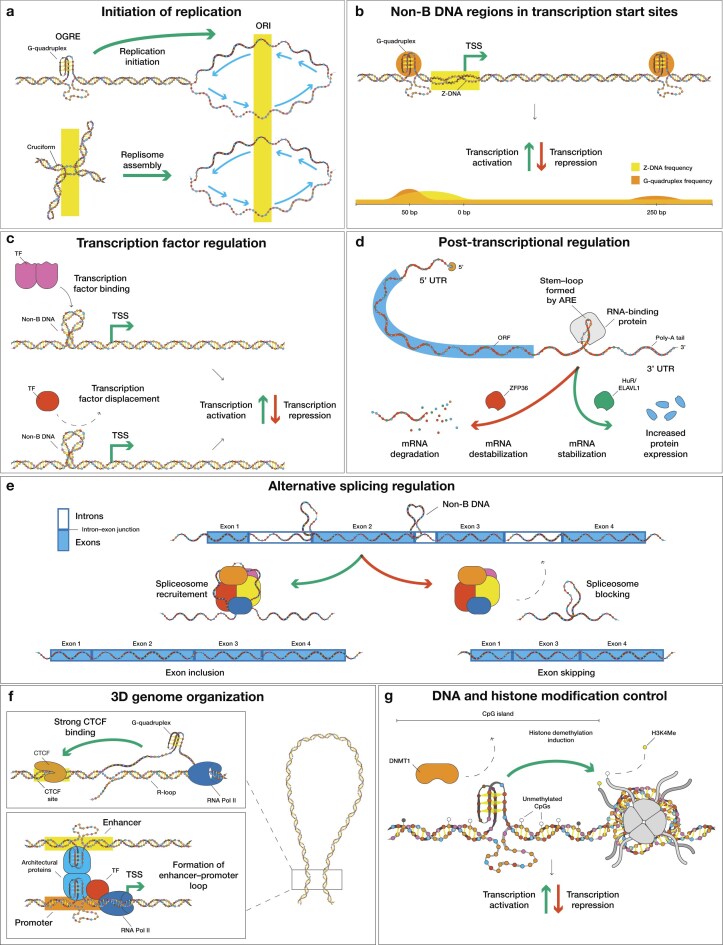
Consequences of non-B DNA structures present at regulatory elements. (**A**) G-quadruplexes located at OGREs promote origin firing and replisome assembly. (**B**) G-quadruplexes and Z-DNA near transcription start sites (TSSs) bias transcriptional initiation and pausing, thereby increasing transcriptional activation. (**C**) Non-B DNA-forming sequences adjacent to transcription factor (TF) binding sites can either recruit or displace TFs. (**D**) Hairpins formed at AU-rich elements (AREs) aid RNA-binding protein (RBP) binding and can either stabilize mRNA or accelerate mRNA decay. (**E**) Non-B DNA conformations near splice junctions alter spliceosome recruitment, driving exon inclusion or skipping. (**F**) R-loops can co-localize with G-quadruplexes, enhancing CCCTC-binding factor (CTCF) binding and supporting long-range enhancer–promoter looping via YY1 and RNA Pol II. (**G**) Non-B DNA motifs within CpG islands can limit DNMT1 activity, leading to hypomethylated states and promoting histone demethylation.

Near or within origins of replication (ORI), non-B DNA-forming sequences can influence replication in two opposing ways: they can activate DNA replication, yet they can also impede polymerase progression [116–118]. On one hand, an origin G-rich repeated element (OGRE), known to form G-quadruplex structures, facilitates replication initiation in mammalian genomes, while its deletion strongly reduces origin firing [116, 119] (Fig. 3A). On the other hand, i-motif structures in the template strand can cause polymerases to stall more efficiently than G-quadruplexes [120]. G-quadruplex and i-motif structures are mutually exclusive in most cases, despite originating from complementary strand contexts. During replication, their formation is regulated by a cell-cycle-dependent “switch”. G-quadruplexes peak during S phase [57], while i-motifs reach their maximum during late G1 phase [62, 121]. Inverted repeats, and especially cruciform-forming sequences, have been found to act as triggering signals for replication initiation, when placed in ORI sites [122] (Fig. 3A). Non-B DNA motifs can also stall polymerase complexes. For example, a Z-form DNA–RNA hybrid, which can be stabilized by CpG methylation, was found to inhibit the initiation and elongation of Okazaki fragments [123]. In mammalian cells, H-DNA-forming repeats can antagonize ORI activation by limiting helicase-driven duplex unwinding. Finally, the H-DNA-induced stalling is more prominent when the non-B DNA-forming sequence is present on the lagging strand [124].

Non-B DNA-forming sequences are also involved in the transcriptional process, and often accumulate near or within promoters, enhancers, insulators [11, 21, 62, 125–132], TSSs, and transcription end sites (TESs) [21, 133–135]. Studies have shown that non-B DNA motifs can regulate gene expression through multiple mechanisms, including controlling transcription factor binding, nucleosome positioning, epigenetic modification regulation, and modulating splicing and translation [11, 21, 62, 125–132, 136–139] (Fig. 3B). Non-B DNA structures within or adjacent to transcription factor binding sites can either aid in transcription factor recruitment and stabilization, or displace transcription factors entirely [16, 21, 139] (Fig. 3C). For example, the interaction of YY1 with G-quadruplexes can aid DNA looping [140] and recognition of non-B DNA structures by p53 [141], whereas reporter plasmid assays show G-quadruplexes within the *KRAS* promoter reduce its transcriptional efficacy by ∼20% [142]. An i-motif-forming sequence present in the mid-region of the *KRAS* promoter is recognized by the transcription factor hnRNP K, which positively modulates *KRAS* transcription [143]. In the *BCL2* promoter, an i-motif structure exists in equilibrium with a hairpin conformation, where the i-motif structure can be detected by hnRNP LL, leading to transcriptional activation [144, 145]. The presence of i-motif sequences in the *MYC* promoter was shown to diminish gene expression [146], whereas G-quadruplex formation has shown mixed outcomes. For instance, a recent study demonstrated that CRISPR-dCas9 fused to nucleolin targeting the *MYC* P1 promoter G–quadruplex-forming sequence effectively induced G-quadruplex folding, resulting in transcriptional repression of *MYC* [147]. Another study genetically abrogated endogenous G-quadruplex structure formation at the *MYC* P1 promoter, resulting in reduced expression [148]. G-quadruplexes can also mediate distant enhancer–promoter interactions. Using a CRISPR-Cas9 system, Doyle *et al.* (2025) demonstrated that G-quadruplex-forming sequences present in the α- and β-globin enhancer loci are necessary to sustain active chromatin, RNA Pol II recruitment, and enhancer–promoter interactions. Consistent with these findings, disruption of the G-quadruplex-forming sequence hinders globin transcription, whereas replacement with an unrelated G-quadruplex-forming sequence restores enhancer function [149]. Z-DNA structures can have a dual effect on transcription regulation. On one hand, Z-DNA-forming sequences have been found to drive increased expression when placed upstream of genes [21]. On the other hand, *in vitro* results have shown that the RNA polymerase can partially stall at the B-Z junction when moving from a B-DNA segment to a Z-DNA segment [124, 150]. H-DNA formation can also impede transcription. During RNA polymerase elongation, additional negative supercoiling is generated behind the enzyme, promoting non-B DNA formation at H-DNA-forming sequences. This in turn creates a sharply bent DNA structure that can lead to polymerase stalling [151].

AREs are the most common *cis*-regulatory elements located within 3′ untranslated regions (UTRs), and are capable of hairpin structure formation [152–154]. Such hairpin structures are recognized by RBPs and play a crucial role in regulating messenger RNA (mRNA) decay at the post-transcriptional level. HuR/ELAVL1 and ZFP36 are two examples of RBPs that bind hairpin AREs and function in opposite ways. HuR/ELAVL1 is a mRNA stabilization factor that antagonizes mRNA decay by blocking access of destabilizing factors, limiting recruitment of the decapping machinery, leading to increased protein output [155–158] (Fig. 3D). In contrast, ZFP36 recognizes the same hairpin AREs, but recruits the CCR4-NOT deadenylase to drive mRNA decapping, resulting in faster decay and minimized protein expression [159–163] (Fig. 3D). Transcriptome-wide PAR-CLIP analysis revealed that over 80% of ZFP36 sites in 3′ UTRs overlapped with HuR/ELAVL1 target sites. ZFP36 exhibited higher affinity for AREs compared to HuR/ELAVL1 [164], and *in vitro* models show that ZFP36 has very low basal expression, with HuR/ELAVL1 being more abundant in cells. Alternative pathways for 3′ UTR hairpin-mediated mRNA decay have also been identified [160]. Finally, non-B DNA-forming sequences are enriched near splice junctions, exhibiting either recruitment or repulsion of splicing factors [165–167] (Fig. 3E).

The inherent genomic instability of non-B DNA can facilitate biological processes such as gene regulation and recombination. For example, co-transcriptionally formed R-loops have been shown to promote recombination [168, 169] and immunoglobulin class switch recombination [170]. Z-DNA-forming sequences serve as markers for the AIRE transcription factor to select genes that drive thymic T cell tolerization, through DNA double-strand break (DSB) formation and promoter poising [136]. G–quadruplexes in immunoglobulin switch regions have been shown to enhance AID targeting, thereby promoting class–switch recombination [171, 172]. Non-B DNA-forming sequences are also enriched at recombination sites, including at binding sites of PRDM9, a protein responsible for specifying the genomic sites of meiotic recombination in mammals [173–176]. H-DNA-forming sequences have also been shown to induce homologous recombination in mammalian cells, with H-DNA-forming oligonucleotides often discussed as potential genome editing tools [177–179]. In addition, cruciform-forming sequences can lead to DNA rearrangements by both replication-dependent and -independent mechanisms [23].

Non-B DNA can also contribute to higher-order genome organization and epigenetic regulation. G-quadruplexes on the G-rich strand can facilitate the formation of R-loops on the complementary strand at CTCF sites, promoting CTCF binding and hence genome architecture organization [130, 180–182] (Fig. 3F). Attenuating R-loop formation has been shown to reduce CTCF occupancy and alter gene expression. G-quadruplex-forming sequences are also enriched at topologically associated domain boundaries [183]. Non-B DNA structures help bridge distal elements. As previously mentioned, the presence of G-quadruplex-forming sequences in enhancers and promoters facilitates identification by YY1, and subsequent YY1 dimerization and mediation of long-range enhancer–promoter looping [184] (Fig. 3F). It has been proposed that enhancers and promoters can form G-quadruplexes cooperatively, with each region contributing “half of a G-quadruplex”, leading to distant DNA looping without the need for architectural proteins [185, 186]. G-quadruplex structures identified within CpG islands have been found to inhibit DNMT1 enzymatic activity, leading to local hypomethylation [187, 188] (Fig. 3G). R-loop formation in promoter CpG islands likewise protects against DNA methylation from DNMT3B [131, 189], and both *in vitro* and *in vivo* studies suggest that Z-DNA structures inhibit DNMT3A [190, 191]. Furthermore, promoter G-quadruplex-forming sequences and associated proteins (i.e., NME2) can recruit the REST-LSD1 complex and induce histone H3 Lys4 demethylation [9] (Fig. 3G). Other non-B DNA-forming sequences have been found to affect nucleosome placement. *In vitro* and *in vivo* results show that G-quadruplex-forming sequences are located at the deepest point of nucleosome exclusion at promoters and correlate with maximum promoter activity [192]. H-DNA formation is thought to be inhibitory to nucleosome assembly, leading to a bias toward H-DNA exclusion from nucleosomes [193]. Z-DNA-forming sequences can also be refractory to nucleosome assembly [194], and hence they can not be incorporated within histone cores, except at their termini [195]. However, these inhibitory effects are not universal across non-B DNA-forming sequences; some long triplet repeat sequences, that can adopt hairpin structures, have instead been found to be strong nucleosome positioning elements [193].

Because non-B DNA-forming sequences occur in repetitive genomic regions, they are often found in telomeric and centromeric loci. Telomeric TTAGGG tracks primarily adopt T-loop formation. During replication or TERRA transcription, these T-loops are broken and can form G-quadruplexes, i-motifs, or R-loops, which assist in telomere capping and maintenance. However, if these non-B DNA structures are not properly resolved, they can induce DNA damage leading to recombination-based telomere lengthening and end-end fusions [196–199]. Telomere-to-telomere genomic assemblies of six great apes have shown substantial enrichment for non-B DNA-forming sequences in centromeric regions, with inverted repeats displaying moderate enrichment at approximately half of all centromeres analyzed [200, 201]. It is also proposed that centromeres form at non-B DNA motifs and can be stabilized by sequence-specific DNA-binding proteins [40, 202].

## Non-B DNA across the tree of life

Non-B DNA-forming sequences are often enriched at functionally important genomic sites, though differences can be found across the tree of life. Analysis of more than 100,000 genomes showed that G-quadruplex-forming sequences are unevenly distributed across the three domains of life and viruses, with lineage-specific preferences. Despite this variability, their genomic placement is nonrandom, with *cis*-regulatory elements exhibiting higher enrichment. For example, in bacteria belonging to the Deinococcota phylum, G-quadruplexes are preferentially placed upstream and downstream of TSSs and TESs, respectively [108]. Near splice sites, G-quadruplex motifs are predominantly found in certain lineages, such as vertebrates and birds, but are depleted in others [165]. Across great apes, G-quadruplexes are also enriched at enhancers, promoters, UTRs, and ORI, but depleted at protein-coding regions. Also, short tandem repeats are enriched in the repetitive telomeric and centromeric regions [201]. For both motifs, additional species-specific enrichments are observed [203]. In yeast, transcription-replication head-on collisions during meiosis lead to de novo R-loop formation, which may promote meiotic DSBs [204].

Cruciform/hairpin-forming inverted repeats are the most abundant non-B DNA promoter motif in prokaryotes [205, 206], while in the case of *Vibrio parahaemolyticus*, a pathogenic *Vibrio* species, cruciform elimination by the HlyU protein enables concomitant virulence gene expression [207]. Inverted repeats can also drive Rho-independent transcription termination and are often found in terminator regions [208]. In bacterial biofilms, G-quadruplexes and Z-DNA protect from nuclease degradation [209]. Across most viral species, G-quadruplexes are associated with repeats, replication origins, and 5′ and 3′ UTRs. The *Maculavirus vitis* (Grapevine fleck virus) is the most G-quadruplex-rich organism found to date (≈23.8 putative G-quadruplexes per 1000 nt, 66% of its genome is covered by G-quadruplex-forming sequences) [210]. This extreme enrichment is the result of a very strong GC-skew (uneven asymmetry G versus C distribution between the two strands): the complementary negative (–) replication intermediate is extremely G-rich and forms long G-runs, which dramatically favors the G-quadruplex abundance. In contrast, G-quadruplexes tend to be detrimental to SARS-CoV-2 by creating steric hindrance for the viral RNA polymerase during replication and by blocking ribosome elongation [211]. At the same time, hairpin formation at the 5′ UTR promotes viral transcription [212]. Finally, Z-DNA acts as an innate immune cue via Zα-domain-containing proteins. Host cytosolic ZBP1 binds to viral Z-nucleic acids generated during replication or transcription and can trigger necroptosis and inflammatory signaling [213].

Non-B DNA motifs are abundant in repetitive regions in genomes and represent the cumulative outcomes of evolutionary processes, serving as molecular records of genomic history. Since non-B DNA structures can stimulate genetic instability, they can initiate genomic variation [21, 174, 214–216] and provide targets for selection and therefore are considered as drivers of genome plasticity and evolution [217–221]. For example, direct and inverted repeats in the terminal inverted repeats of common transposable elements can stimulate the formation of DSBs, which can lead to gap-filling and capturing of nearby sequences as fillers, or cause template switching [222]. Through these steps, transposable elements could amplify and spread themselves within genomes. Repetitive elements in centromeric and telomeric repeats can also stimulate the formation of DSBs and mediate recurrent chromosome fusions, drastically reducing the chromosome numbers; for example, from 2n = 70 in the ancestral karyotype to 2n = 6/7 in *M. muntjak vaginalis* [223]. Further, Z-DNA-forming sequences have been shown to stimulate recurrent deletions of a pelvic enhancer of the *Pitx1* gene, resulting in many independently derived freshwater stickleback fish that adaptively lost pelvic hind fins in <15,000 years [224]. Structurally complex DNA sequences containing G-quadruplex, H-DNA, and hairpin motifs have rapidly expanded in eumetazoan genomes, indicating their potential evolutionary utility [225]. Their over-representation at promoters, enhancers, UTRs, and other functional elements positions mutation-prone DNA to be both a likely driver of genomic variation and often under selection constraints [226–228], is involved in aging, and a contributor to human diseases, including cancer.

## Higher mutation rates at non-B DNA loci in germline and somatic contexts

Experimental systems have been instrumental in uncovering the mechanisms underlying the instability of non-B DNA-forming sequences. Genomic instability has been observed *in vivo* and in cultured cells for a number of non-B DNA-forming sequences, including inverted repeats [23], Z-DNA [24, 229], and H-DNA [22, 32]. In addition, non-B DNA motifs are detected more frequently in cancer cells than in normal cells [230]. While mutations can contribute to aging and many human diseases, the role of non-B DNA structures as drivers of these mutations through genomic instability is only partially understood [33, 231–233]. Systematic analysis of mutations from healthy and cancer genomes has provided critical evidence for the contribution of non-B DNA-forming sequences to germline and somatic mutagenesis [19, 31, 214, 216]. Base substitutions, insertions and deletions (indels), and structural variants/rearrangements have all been found enriched at non-B DNA loci [19, 31, 215, 231]. Different non-B DNA categories show higher enrichment for specific mutation types and under particular conditions or contexts.

**Figure 4.**
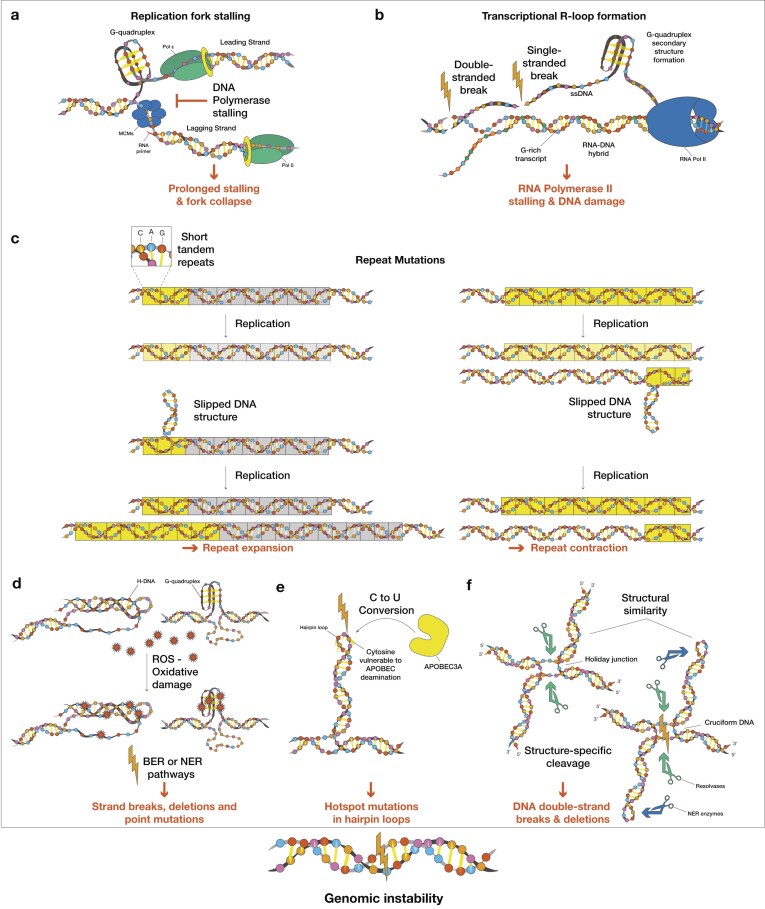
Non-B DNA structure formation drives replication and transcription-associated genomic instability. (**A**) G-quadruplexes can cause replication fork stalling, leading to fork collapse and DSBs. (**B**) R-loops formed during transcription can block RNA Pol II progression and promote DNA breaks. (**C**) Short tandem repeats adopt slipped structures during replication, resulting in repeat expansions or contractions. (**D**) H-DNA and G-quadruplexes accumulate more oxidative DNA damage than B-DNA, which can interfere with repair processes, predisposing sequences to mutations. (**E**) Hairpin loops are susceptible to APOBEC3A-mediated cytosine deamination, generating hotspot mutations. (**F**) Cruciform structures form Holliday junction-like intermediates that undergo structure-specific cleavage, causing strand breaks and deletions.

Several non-B DNA structures can obstruct both the transcription and the replication machinery, leading to DNA and RNA polymerase stalling, fork collapse, and genomic instability [23, 150, 151, 234–240]. Beyond direct polymerase stalling, non-B DNA-forming sequences can also increase the probability of replication-transcription collisions when the two processes happen on the same DNA template. These conflicts can happen either head-on, when the replisome and the transcription complex approach one another from opposite directions, or co-directionally when a replication fork catches up to the transcription complex moving in the same direction, due to transcription stalling from a non-B DNA structure [124]. During replication, G-quadruplexes can form spontaneously on the leading-strand template within the newly unwound DNA region, directly behind the minichromosome maintenance helicase (MCM) and before nascent DNA strand synthesis (Fig. 4A). These G-quadruplexes can locally perturb replisome organization, leading to reduced DNA synthesis [241]. Similar replisome stalling has been observed *in vitro* for i-motifs, which can lead to fork collapse and DNA deletions [14, 242, 243]. R-loops can also impede transcription and replication [244–247]. For example, they can form behind RNA Pol II, where negative supercoiling unwinds the DNA duplex, allowing the 5′ end of the nascent RNA to hybridize with the template strand. In G-rich regions, this is accompanied by G-quadruplex formation in the non-template strand [51, 248] (Fig. 4B). An H-DNA-forming sequence present in the human *MYC* promoter has been found to stall replication forks, leading to the formation of DSBs [124]. Cruciform-forming inverted repeats likewise impede fork progression and can be processed by structure-specific nucleases (e.g., ERCC1–XPF). When cleaved, they can also lead to DSB formation and large deletions [249]. It is important to note that such outcomes are not strictly replication dependent. For example, H-DNA-, Z-DNA-, and cruciform-forming sequences can stimulate DSBs even in replication-deficient extracts, consistent with repair-driven mutagenesis that can occur in the presence or absence of replication [250].

Several specialized helicases have been found to resolve non-B DNA conformations, which, if left unresolved, can lead to DNA damage and mutagenesis [234, 235, 251–253]. To characterize such molecular interactions, X-ray crystallography [254, 255] and cryo-EM [256, 257] have been employed, revealing that they occur via selectively distinct interaction surfaces, which differ from those observed for duplex DNA. G-quadruplexes generated during replication are primarily resolved by FANCJ, BLM, and WRN, among others [258–261]. Evidence from model organisms suggests that in the absence of these helicases, G-quadruplexes can persist throughout the cell cycle, promoting genomic instability [252, 262]. Helicase depletion can increase fork stalling, DSBs, and rearrangements at G-rich loci [263]. R-loop resolution is also regulated by a network of specialized enzymes, including BRCA1, SETX, and TOP1, and their deficiency results in R-loop build-up [264–267]. Persistent R-loops can cause replication-transcription conflicts that generate mutations, DSBs, and large-scale chromosomal rearrangements [245, 268–271].

H-DNA is also found to have a broad interactome of triplex-associated proteins. DDX3X directly unwinds H-DNA, thereby preventing double-strand DNA breaks at H-DNA loci [272]. DHX9 has also been found to resolve H-DNA conformations *in vivo* and *in vitro*, and its absence leads to increased genomic instability and mutagenesis [273]. Both helicases also unwind G-quadruplexes. They resolve transcriptionally formed G-quadruplex structures that would otherwise lead to polymerase stalling [274, 275], as well as G-quadruplexes that stall ribosomes, which would in turn lead to depleted protein synthesis and cell senescence [276, 277]. These findings suggest that these helicases are not restricted to a single non-B DNA structure but are promiscuous to multiple non-B DNA types. In addition to causing replication stress, non-B DNA structures are frequently misrecognized by repair proteins as DNA lesions, thereby activating repair pathways, or as ssDNA resembling invading viral genomes, thereby triggering mutagenesis [250, 278, 279]. Such misprocessing can also interfere with the recognition and repair of DNA damage, leading to error-prone outcomes [280–282]. In addition, some non-B DNA-forming sequences can stimulate damage response pathways [124, 283, 284].

Replication fork stalling at unresolved non-B DNA structures can generate stretches of ssDNA coated with replication protein A (RPA), which can trigger ATR recruitment and Chk1 phosphorylation to activate cell cycle checkpoints [285]. When stalling progresses to fork collapse or the formation of DSBs, ATM-Chk2 signaling occurs, driving γH2AX formation and the formation of DNA damage foci [286]. In parallel, PARP1 acts as a sensor for both DNA damage and non-B DNA structures [287]. Upon binding to G-quadruplexes, R-loops, and cruciforms, PARP1 becomes catalytically active and synthesizes PAR chains, which recruit base excision repair proteins, single-strand break/DSB repair factors, and modulate chromatin structure [288–290]. Finally, G-quadruplex formation has been shown to recruit the Ku70/80 heterodimer to DSBs, which in turn recruits DNA-PKcs to the sites. The following phosphorylation of DNA-PKcs is crucial for the induction of DNA repair mechanisms [282]. Different DNA damage response pathways are associated with genomic instability across non-B DNA categories and are described in detail by Wang and Vasquez [6].

In addition to activating damage responses, repetitive non-B DNA sequences are intrinsically mutagenic through replication-dependent mechanisms. Direct and short tandem repeats can cause strand slippage events, resulting in frequent indels and substitutions [291] (Fig. 4C), a process that is intensified when mismatch repair is compromised. Unstable microsatellites undergo similar processes, producing a phenotype often observed in cancer [292]. Strand slippage events also occur at other non-B DNA types that coincide with repetitive sequences [19]. Trinucleotide repeat instability is a critical phenomenon underlying many neurological and other genetic disorders [232, 293, 294]. CAG repeats can form slipped DNA and hairpin structures, whose expansion or contraction depends on replication directionality [293]. Similarly, pentanucleotide repeats identified within introns have been associated with a variety of neurodegenerative diseases [295, 296]. Short tandem repeat sequences prone to forming slipped structures undergo expansions that can drive somatic instability, creating a random process of repeat length variation across tissues [297]. The structural instability and/or error-generating processing of these expanded repeats underscore the complex relationship between repeat-expansion dynamics and modulation of repair pathways. Finally, genome-wide analyses show that non-B DNA-forming sequences present in intergenic regions exhibit higher mutation densities than matched B-DNA controls [214, 228].

Non-B DNA-forming sequences are enriched at substitution sites, indel sites, and translocation breakpoints across cancer genomes [19, 31, 298], and have further been linked to copy number variation hotspots [299]. Some structure-forming sequences, such as G-quadruplexes, also coincide with oxidative DNA damage hotspots, particularly 8-oxo-guanine lesions leading to abasic sites and strand breaks [300, 301]. H-DNA-forming sequences also accumulate more oxidative lesions than B-DNA under conditions of oxidative stress [302] (Fig. 4D). In the human genome, thermodynamically stable inverted repeats with long arms, free of mismatches, are largely absent [303–306]. Experiments in which long inverted repeats are artificially introduced in eukaryotic cells show that these are genomic instability hotspots, which are lost within a few cell cycles [307]. Hairpin/cruciform-forming inverted repeats with long arms containing mismatches do exist, and they are linked to DSBs and chromosomal rearrangements [308–310]. In cancer genomes, inverted repeats are a mutational hotspot in part due to APOBEC enzyme off-target activity [216, 311, 312], but also via other mutagenic processing mechanisms, leading to the formation of DSBs and indels [19, 23] (Fig. 4E). It was also shown that functionally significant cancer “driver” mutation identification can be convoluted by the elevated mutation rates at pre-existing non-B DNA-forming sequences, which create recurrent mutational hotspots independent of selective pressure [19, 216]. Finally, inverted repeats are hotspots for DSBs and deletions in mammalian cells. Cruciforms formed at inverted repeat sequences resemble Holliday junctions in their four-way DNA structure, and can be recognized by resolvases (i.e., SLX1–SLX4, MUS81–EME1, GEN1) and nucleotide excision repair enzymes (e.g., XPF–ERCC1), resulting in genetic instability [23, 313] (Fig. 4F).

Additionally, stabilization of non-B DNA structures with ligands increases DNA damage responses and genomic instability, due to polymerase stalling, helicase inhibition, and aberrant repair. By stabilizing them with ligands, they act as roadblocks to stalling both RNA Pol II during transcription and Pol *ε* and *δ* during replication, leading to transcription-replication conflicts or replication fork collapse and finally the formation of DSBs [283, 314, 315]. The increased stability of G-quadruplexes in the presence of molecules such as PhenDC3 and TMPyP4 counteracts the activity of G-quadruplex-resolving helicases, enhancing previously mentioned events associated with genetic instability [283, 316]. G-quadruplex stabilization in telomeres has been shown to induce dissociation of the telomere-capping protein TRF2 and has also been found to prevent the formation of protective T-loop structures. This hinders repair in telomeres, and the effects can be expressed as sister telomere fusions and telomere loss [317, 318]. The effects of non-B DNA stabilizing molecules on genetic instability can be detected by immunofluorescence of γH2AX, p53BP1, pATM foci, ChIP-seq analysis of γH2AX genome-wide sites, and comet assays [269, 315].

While experimental studies provide mechanistic insights into key loci, computational analyses extend these observations to the genome-wide level. Whole-exome and whole-genome sequencing mutation datasets of healthy individuals and of tumor samples have offered valuable insights into the global impact of non-B DNA motifs. However, their interplay with various mutagen exposures and the presence/absence of DNA repair pathways remains only partially understood. Incorporating mutational signature analysis may help us better understand these complex interactions [311, 319] thus, future work in this direction is warranted

## Biophysical determinants of non-B DNA-associated mutagenesis

**Figure 5.**
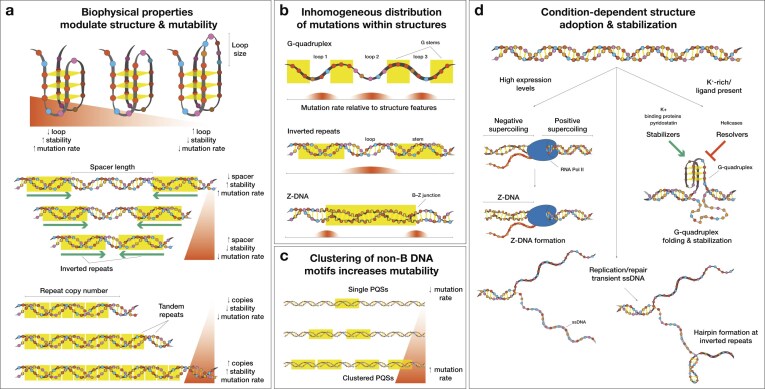
Biophysical characteristics of non-B DNA structures directly influence associated genomic instability. (**A**) Structural features such as loop and spacer length modulate non-B DNA motif stability, with shorter loops or spacers exhibiting higher mutation rates. (**B**) Non-B DNA structures expose ssDNA that accumulates substitution mutations at loops of G-quadruplexes and inverted repeats and at B-Z junctions of Z-DNA. (**C**) Clusters of non-B DNA motifs generate locally elevated mutational burden. (**D**) The cellular environment directly influences the structural conformation of a nucleotide sequence. PQSs: Potential G-quadruplex sequences

As described previously, non-B DNA conformations contribute to localized increases in mutation rates. These effects are directly shaped by their biophysical and sequence properties, which affect the formation and stability of non-B DNA structures. Biophysical characteristics promoting more frequent or more stable non-B DNA formation are associated with heightened mutability, whereas features that destabilize them, including mismatches, reduce the observed mutation rates [19, 215, 216, 311, 320, 321]. G-quadruplexes with smaller loops form more readily, exhibiting higher mutation frequencies, as do inverted and direct repeats with short spacer lengths [19, 311, 320] (Fig. 5A). Using an APOBEC cytidine deamination activity assay, it was shown that biophysical properties of inverted repeats, including the spacer and arm lengths as well as the nucleotide composition, influence the levels of deamination activity. Tandem repeats become increasingly unstable as copy number grows [322, 323]. Mismatches within the repeat unit reduce mutability by destabilizing secondary structure formation [324–327] (Fig. 5A). Both repeat unit sequence and copy number are the primary determinants of tandem repeat formation across loci. Non-B DNA structures expose ssDNA, which is prone to damage and mutagenesis [19, 216, 311]. For instance, the exposed loops of G-quadruplexes [19, 174, 215] and of inverted repeats show an excess of substitutions [19, 216, 311], whereas Z-DNA accumulates mutations closer to the B-Z junction [215] (Fig. 5B). Clusters of non-B DNA structures result in even higher locally elevated mutation rates [315, 328, 329] (Fig. 5C). Depending on the cellular milieu, similar sequences can adopt different non-B DNA structures, with conditions that favor stabilization driving increased mutagenesis [269, 315, 329–335] (Fig. 5D). For example, Z-DNA forms under negative supercoiling caused by high transcription rates, inverted repeats generate transient hairpins during replication or repair, and K^+^ ions act as stabilizers for G-quadruplexes. Additionally, small-molecule G-quadruplex stabilizers are consistent drivers of increased DNA damage [269, 315].

## Non-B DNA functional exemplars at cancer gene promoters

Studies focusing on cancer gene promoters have shown that non-B DNA-forming sequences coincide with mutational hotspots and can significantly change the expression levels of downstream genes. Multiple non-B DNA motifs were found in the promoter of the oncogene *MYC*, including G-quadruplex, i-motif, H-DNA, and Z-DNA conformations [148, 336–340]. Mutational disruption of the G-quadruplex leads to transcriptional loss, whereas small-molecule stabilization suppresses it [148, 340–342]. In the *BCL2* promoter, multiple non-B DNA structures can be formed and have been experimentally tested for their regulatory roles [336, 343–345]. For example, G-quadruplexes formed in the P1 promoter are stabilized by oxidative lesions and can shift the equilibrium between B-DNA and G-quadruplex structures, potentially altering *BCL2* transcription [346]. The *KRAS* promoter harbors G-quadruplex sites, which regulate expression levels and coincide with mutational hotspots [142, 347]. The *TERT* promoter is one of the most studied noncoding drivers across different cancer types [348]. Multiple mutational hotspots at this locus overlap with G-quadruplex loci, and the recurrent mutations identified disrupt both the G-quadruplex sequence and key transcription factor binding sites [349, 350]. Studies of the promoters mentioned above have shown that modulating the kinetics of non-B DNA conformation in these loci with small molecules can change the expression of downstream genes [333, 344, 351, 352], indicating their potential for drug discovery. Specifically, by manipulating the thermodynamic stability and interfering with the resolution of non-B DNA structures at target genes, new candidate drugs can be developed [353, 354]. Interestingly, recurrent repeat expansions have been observed across different cancer types, with most being cancer-specific, and a subset of these was shown to result in a dose-dependent decrease in cell proliferation [355].

## Interpreting recurrence: drivers versus passenger mutations

Not all recurrent mutations in non-B DNA-forming sequences are consequential for cancer development. The prevailing dichotomy categorizes mutations as either drivers, which directly contribute to cancer development, or passengers, which are considered incidental byproducts of the genomic instability inherent to cancer cells [356]. Mutation recurrence has long been considered an indicator of positive selection. Understanding the determinants of the mutation rate variation in the human genome is pivotal for developing sensitive statistical models that can accurately detect selection in cancer.

Previous research has shown that recurrent mutations are more likely to overlap with non-B DNA sequences than expected by chance [19]. However, this might be driven by heightened genomic instability, selection, or both. Passenger mutation hotspots at inverted repeats in cancer-associated genes are often driven by APOBEC3A off-target mutagenesis [216]. For instance, recurrent mutations at the *PLEKHS1* promoter, observed across multiple cancer types, occur within the spacer regions of inverted repeats and are attributed to APOBEC activity [321, 349, 357]. Nonetheless, their impact on gene expression remains inconclusive, as some studies report significant effects, while others do not [321, 349, 358]. Other inverted repeat mutation hotspots have also been characterized, with models developed to predict their mutability [216, 311]. These models account for the biophysical properties of the hairpin structure, including spacer and arm lengths as well as the nucleotide composition of each, which together influence the thermodynamic stability. In these cases, the heightened rate of mutagenesis is a more likely explanation than selection during cancer development.

Finally, the contribution of non-B DNA structures in driving human disease may be two-fold and context-dependent. First, noncanonical DNA conformations can have functional consequences, such as influencing gene expression, splicing, and translation. Secondly, their presence increases the likelihood of mutagenesis at a given site, which in certain cases can contribute to human disease and cancer development, while in others might have little or no effect. To investigate the impact of these mutations, examining the distribution of mutations within the sub-components of non-B DNA motifs and their surrounding regions, along with analyzing the biophysical properties of the motif and the associated mutational processes, can provide valuable insights. Further experiments are needed to elucidate their specific contributions and underlying mechanisms, which can differ from one case to another.

## Non-B DNA as drivers of aging and tissue dysfunction

Non-B DNA loci might be pivotal for evolutionary adaptation, as they can accumulate mutations faster than the rest of the human genome and increase genetic variation at the population level. However, the same selection forces can act in somatic cells, resulting in clonality and tissue dysfunction. In somatic cells, the heightened genomic instability at non-B DNA loci can therefore drive human diseases and aging. Loss of the RecQ helicases, which drive premature aging syndromes, disrupts transcriptional regulation at G-quadruplex loci, linking G-quadruplex metabolism to age-associated gene expression changes [359]. WRN deficiency is also found to have a two-fold increase in mutational frequency, both for single base substitutions and deletions at H-DNA and Z-DNA-forming sequences [360].

In Alzheimer’s disease, hippocampal DNA from severely affected patients exhibits strong B-to-Z-DNA transitions. Z-DNA-forming sequences in the promoters of the *APP, APOE*, and *Presenilin* genes may alter their transcription and contribute to disease progression [361, 362]. In Huntington’s disease, long expansions of CAG repeats in the *HTT* gene progressively exceed a toxic threshold in vulnerable neurons, with expansion rates accelerating with age and driving neurodegeneration [363]. In amyotrophic lateral sclerosis and frontotemporal dementia, the GGGGCC hexanucleotide repeat expansion found in *C9orf72* has been found to form G-quadruplexes and R-loops impeding RNA polymerase transcription, which results in transcriptional pausing and abortion [364].

Studies in model organisms have revealed tissue-specific, age-dependent increases in non-B DNA-associated mutagenesis. Age-related mutagenesis at H-DNA motifs from the human *MYC* gene increases in mouse spleen but not in testis [35]. Similarly, cruciform-induced genomic instability increased mutation frequencies in aging spleen and brain tissues of mice, compared to B DNA [365]. Additionally, H-DNA-induced mutagenesis has been found to increase with age in the mouse spleen and liver, and decrease in the brain [37]. In *Drosophila melanogaster* photoreceptor neurons, R-loop levels increased with age, and their accumulation across long, highly expressed genes disrupted gene expression and visual function. Notably, enhancing R-loop resolution via overexpression of RNase H1 or Top3β rescued visual decline [366].

Mitochondrial DNA (mtDNA) is also susceptible to non-B DNA-mediated instability. Deletions in mtDNA accumulate in aging tissues and can contribute to neurodegeneration, with hairpin- and cruciform-forming sequences significantly enriched at deletion breakpoints. G-quadruplex structures in mtDNA potently stall Pol γ *in vitro*, and analysis of human population cohorts reveals significant enrichment of variants within G-quadruplex-forming regions, suggesting these structures promote mutagenesis during replication [367–369].

Interestingly, non-B DNA-induced mutagenesis does not always increase with age, though non-B DNA-associated DSBs increase as well as apoptosis, which can also contribute to tissue dysfunction and aging [36]. Thus, overall, the faster accumulation of mutations at non-B DNA loci relative to B-DNA, and the increase in DSBs and apoptosis with age in somatic cells can account for a disproportionate contribution to aging. These emerging connections between non-B DNA-forming sequences and aging warrant further investigation. Population-level analyses of healthy aging individuals across tissues can help identify non-B DNA loci that predispose certain organs to age-related dysfunction. A deeper understanding of non-B DNA-mediated mutagenesis in aging may inform strategies to preserve genomic integrity and extend human healthspan.

## Conclusions and future directions

Recent advances in long-read sequencing technologies have expanded our capacity to study non-B DNA-forming sequences. Long-read sequencing services from Oxford Nanopore Technologies [370, 371] and PacBio’s HiFi [372] allow for the generation of high-quality gapless reference genomes, as evidenced by the work performed by the Telomere-to-Telomere and Human Pangenome Reference consortia [373–375]. Non-B DNA-forming sequences are abundant in repetitive regions of the human genome [173, 201, 202], and human pangenome assemblies enable genome-wide analysis of non-B DNA motif distribution across previously missing repetitive regions [373, 375]. These technologies allow for the simultaneous readout of epigenetic modifications, which can be integrated with non-B DNA analysis, since formation kinetics and structure stability are directly influenced by methylation levels [187, 188, 376, 377]. Increased methylation levels destabilize G-quadruplexes [378], but aid Z-DNA formation [379]. Previously, elevated sequencing errors have been detected at non-B DNA-forming sequences [380, 381], because these sequences are often repetitive and deviate from the standard B-DNA conformation, suggesting that the higher mutation rates in non-B DNA regions may be attributed to sequencing artifacts. The new long-read sequencing technologies have significantly reduced sequencing error rates [382] and enable re-evaluation of these claims, revealing a consistent increase in mutation rates at G-quadruplex regions [174], consistent with previous experimental systems [22, 24, 383, 384].

These technological advances position the field to identify potential roles of non-B DNA-forming sequences in previously inaccessible genomic regions, particularly in the context of lineage-specific genome evolution and genetic diversity. Initial work in this area, utilizing high-quality genome assemblies of primates, has catalogued thousands of predicted non-B DNA motifs in repetitive regions [173, 201, 203].

Another key unanswered question is how non-B DNA structures contribute to somatic mutation accumulation and clonal expansion during aging. Ultra-deep, single-molecule sequencing technologies enable the detection of rare somatic mutations, providing the resolution to quantify low-frequency variants that accumulate in somatic cells during aging and in human diseases [385–389]. Paired with single–nuclei techniques for non-B DNA motif detection, they can reveal cell-to-cell heterogeneity [390]. Application of these technologies could reveal how noncanonical DNA structures contribute to mutational hotspots, clonal expansions, and age-associated genome instability, thereby uncovering links between DNA conformation, mutation accumulation, and the etiology of aging-related diseases.

**Figure 6.**
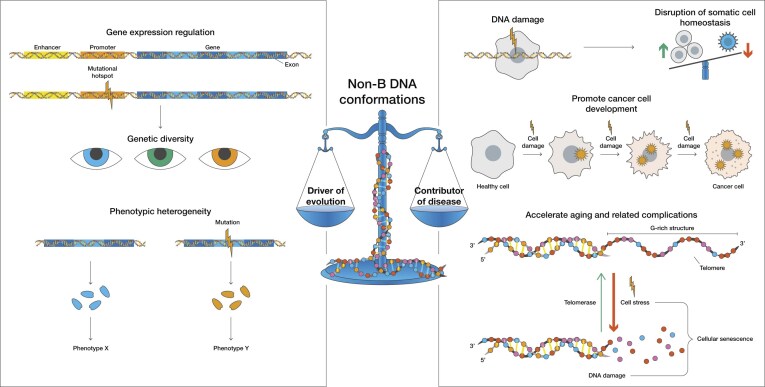
Noncanonical DNA structures act as functional elements but can also be sources of genomic instability. At *cis*-regulatory elements such as promoters, enhancers, and splice sites, non-B DNA motifs modulate gene expression and create localized mutational hotspots that foster genetic diversity and phenotypic heterogeneity. When unresolved, they cause DNA damage, disrupt homeostasis, promote aging, and contribute to the etiology of human diseases.

In conclusion, non-B DNA structures play a dual role in human biology: they are key contributors to genetic variation and evolutionary adaptation, yet they also drive genomic instability that underpins somatic mutagenesis, tissue dysfunction, and human disease. The genomic instability found at non-B DNA loci, while promoting diversity and evolutionary potential, can also accelerate the accumulation of somatic mutations that compromise cellular function, ultimately contributing to aging and diseases (Fig. 6).

## Data Availability

No new data were generated or analysed in support of this research.

## References

[1] Watson JD, Crick FH. Molecular structure of nucleic acids; a structure for deoxyribose nucleic acid. Nature. 1953;171:737–8. 10.1038/171737a0. 13054692

[2] Ghosh A, Bansal M. A glossary of DNA structures from A to Z. Acta Crystallogr D Biol Crystallogr. 2003;59:620–6. 10.1107/s0907444903003251. 12657780

[3] Kaushik M, Kaushik S, Roy K et al. A bouquet of DNA structures: emerging diversity. Biochem Biophys Rep. 2016;5:388–95. 10.1016/j.bbrep.2016.01.013. 28955846 PMC5600441

[4] Choi J, Majima T. Conformational changes of non-B DNA. Chem Soc Rev. 2011;40:5893–909. 10.1039/C1CS15153C. 21901191

[5] Georgakopoulos-Soares IC, Candace SY, Ahituv N et al. High-throughput techniques enable advances in the roles of DNA and RNA secondary structures in transcriptional and post-transcriptional gene regulation. Genome Biol. 2022;23:159. 10.1186/s13059-022-02727-6. 35851062 PMC9290270

[6] Wang G, Vasquez KM. Dynamic alternative DNA structures in biology and disease. Nat Rev Genet. 2022;24:211–34. 10.1038/s41576-022-00539-9. 36316397 PMC11634456

[7] Spiegel J, Adhikari S, Balasubramanian S. The structure and function of DNA G-quadruplexes. Trends Chem. 2020;2:123–36. 10.1016/j.trechm.2019.07.002. 32923997 PMC7472594

[8] Xie KT, Wang G, Thompson AC et al. DNA fragility in the parallel evolution of pelvic reduction in stickleback fish. Science. 2019;363:81–4. 10.1126/science.aan1425. 30606845 PMC6677656

[9] Varshney D, Spiegel J, Zyner K et al. The regulation and functions of DNA and RNA G-quadruplexes. Nat Rev Mol Cell Biol. 2020;21:459–74. 10.1038/s41580-020-0236-x. 32313204 PMC7115845

[10] Ravichandran S, Subramani VK, Kim KK. Z-DNA in the genome: from structure to disease. Biophys Rev. 2019;11:383–7. 10.1007/s12551-019-00534-1. 31119604 PMC6557933

[11] Kouzine F, Wojtowicz D, Baranello L et al. Permanganate/S1 nuclease footprinting reveals Non-B DNA structures with regulatory potential across a mammalian genome. Cell Syst. 2017;4:344–56. 10.1016/j.cels.2017.01.013. 28237796 PMC7432990

[12] Kim N, Jinks-Robertson S. Transcription as a source of genome instability. Nat Rev Genet. 2012;13:204–14. 10.1038/nrg3152. 22330764 PMC3376450

[13] Selvam S, Koirala D, Yu Z et al. Quantification of topological coupling between DNA superhelicity and G-quadruplex formation. J Am Chem Soc. 2014;136:13967–70. 10.1021/ja5064394. 25216033

[14] Williams SL, Casas-Delucchi CS, Raguseo F et al. Replication-induced DNA secondary structures drive fork uncoupling and breakage. EMBO J. 2023;42:e114334. 10.15252/embj.2023114334. 37781931 PMC10646557

[15] Georgakopoulos-Soares I, Parada GE, Hemberg M. Secondary structures in RNA synthesis, splicing and translation. Comput Struct Biotechnol J. 2022;20:2871–84. 10.1016/j.csbj.2022.05.041. 35765654 PMC9198270

[16] Spiegel J, Cuesta SM, Adhikari S et al. G-quadruplexes are transcription factor binding hubs in human chromatin. Genome Biol. 2021;22:1–15. 10.1186/s13059-021-02324-z. 33892767 PMC8063395

[17] Schuster-Böckler B, Lehner B. Chromatin organization is a major influence on regional mutation rates in human cancer cells. Nature. 2012;488:504–7. 10.1038/nature11273. 22820252

[18] Polak P, Karlić R, Koren A et al. Cell-of-origin chromatin organization shapes the mutational landscape of cancer. Nature. 2015;518:360–4. 10.1038/nature14221. 25693567 PMC4405175

[19] Georgakopoulos-Soares I, Morganella S, Jain N et al. Noncanonical secondary structures arising from non-B DNA motifs are determinants of mutagenesis. Genome Res. 2018;28:1264–71. 10.1101/gr.231688.117. 30104284 PMC6120622

[20] Lawrence MS, Stojanov P, Polak P et al. Mutational heterogeneity in cancer and the search for new cancer-associated genes. Nature. 2013;499:214–8. 10.1038/nature12213. 23770567 PMC3919509

[21] Georgakopoulos-Soares I, Victorino J, Parada GE et al. High-throughput characterization of the role of non-B DNA motifs on promoter function. Cell Genomics. 2022;2:100111. 10.1016/j.xgen.2022.100111. 35573091 PMC9105345

[22] Wang G, Vasquez KM. Naturally occurring H-DNA-forming sequences are mutagenic in mammalian cells. Proc Natl Acad Sci USA. 2004;101:13448–53. 10.1073/pnas.0405116101. 15342911 PMC518777

[23] Lu S, Wang G, Bacolla A et al. Short inverted repeats are hotspots for genetic instability: relevance to cancer genomes. Cell Rep. 2015;10:1674–80. 10.1016/j.celrep.2015.02.039. 25772355 PMC6013304

[24] Wang G, Christensen LA, Vasquez KM. Z-DNA-forming sequences generate large-scale deletions in mammalian cells. Proc Natl Acad Sci USA. 2006;103:2677–82. 10.1073/pnas.0511084103. 16473937 PMC1413824

[25] Mellor C, Perez C, Sale JE. Creation and resolution of non-B-DNA structural impediments during replication. Crit Rev Biochem Mol Biol. 2022;57:412. 10.1080/10409238.2022.2121803. 36170051 PMC7613824

[26] Gordenin DA, Lobachev KS, Degtyareva NP et al. Inverted DNA repeats: a source of eukaryotic genomic instability. Mol Cell Biol. 1993;13:5315–22. 10.1128/mcb.13.9.5315-5322.1993. 8395002 PMC360228

[27] Lobachev KS, Rattray A, Narayanan V. Hairpin- and cruciform-mediated chromosome breakage: causes and consequences in eukaryotic cells. Front Biosci. 2007;12:4208–20. 10.2741/2381. 17485368

[28] Maizels N . G4-associated human diseases. EMBO Rep. 2015;16: 10.15252/embr.201540607. PMC4552485 26150098

[29] Bacolla A, Cooper DN, Vasquez KM et al. Non-B DNA Structure and Mutations Causing Human Genetic Disease. John Wiley & Sons, Ltd., 2018, 1–15.

[30] Hannan AJ . Tandem repeats mediating genetic plasticity in health and disease. Nat Rev Genet. 2018;19:286–98. 10.1038/nrg.2017.115. 29398703

[31] Bacolla A, Tainer JA, Vasquez KM et al. Translocation and deletion breakpoints in cancer genomes are associated with potential non-B DNA-forming sequences. Nucleic Acids Res. 2016;44:5673. 10.1093/nar/gkw261. 27084947 PMC4937311

[32] Zhao J, Wang G, Del Mundo IM et al. Distinct mechanisms of nuclease-directed DNA-structure-induced genetic instability in cancer genomes. Cell Rep. 2018;22:1200–10. 10.1016/j.celrep.2018.01.014. 29386108 PMC6011834

[33] Wang E, Thombre R, Shah Y et al. G-Quadruplexes as pathogenic drivers in neurodegenerative disorders. Nucleic Acids Res. 2021;49:4816–30. 10.1093/nar/gkab164. 33784396 PMC8136783

[34] Ibañez K, Jadhav B, Zanovello M et al. Increased frequency of repeat expansion mutations across different populations. Nat Med. 2024;30:3357–68. 10.1038/s41591-024-03190-5. 39354197 PMC11564083

[35] D’Amico AM, Li TT, Vasquez KM. Tissue-specific effects of aging on repeat-mediated mutation hotspots *in vivo*. Biomolecules. 2024;14:1453. 10.3390/biom14111453. 39595629 PMC11592361

[36] Li TT, D’Amico A, Christensen L et al. Effects of aging on Z-DNA-induced genetic instability *in vivo*. Genes. 2025;16:942. 10.3390/genes16080942. 40869990 PMC12385340

[37] D’Amico AM, Li TT, Wylie D et al. Aging alters genomic instability at endogenous mutation hotspots in mice. Sci Rep. 2025;15:36016. 10.1038/s41598-025-20084-9. 41094073 PMC12528765

[38] Vijg J, Dong X. Pathogenic mechanisms of somatic mutation and genome mosaicism in aging. Cell. 2020;182:12–23. 10.1016/j.cell.2020.06.024. 32649873 PMC7354350

[39] Vijg J . From DNA damage to mutations: all roads lead to aging. Ageing Res Rev. 2021;68:101316. 10.1016/j.arr.2021.101316. 33711511 PMC10018438

[40] Makova KD, Weissensteiner MH. Noncanonical DNA structures are drivers of genome evolution. Trends Genet. 2023;39:109–24. 10.1016/j.tig.2022.11.005. 36604282 PMC9877202

[41] Zhao J, Bacolla A, Wang G et al. Non-B DNA structure-induced genetic instability and evolution. Cell Mol Life Sci. 2010;67:43–62. 10.1007/s00018-009-0131-2. 19727556 PMC3017512

[42] Sen D, Gilbert W. Formation of parallel four-stranded complexes by guanine-rich motifs in DNA and its implications for meiosis. Nature. 1988;334:364–6. 10.1038/334364a0. 3393228

[43] Sundquist WI, Klug A. Telomeric DNA dimerizes by formation of guanine tetrads between hairpin loops. Nature. 1989;342:825–9. 10.1038/342825a0. 2601741

[44] Sengupta P, Bose D, Chatterjee S. The molecular tête-à-tête between G-quadruplexes and the i-motif in the human genome. ChemBioChem. 2021;22:1517–37. 10.1002/cbic.202000703. 33355980

[45] Watson J, Hays F, A H et al. Definitions and analysis of DNA holliday junction geometry. Nucleic Acids Res. 2004;32:3017–27. 10.1093/nar/gkh631. 15173384 PMC434437

[46] Brázda V, Laister RC, Jagelská EB et al. Cruciform structures are a common DNA feature important for regulating biological processes. BMC Mol Biol. 2011;12:33. 10.1186/1471-2199-12-33. 21816114 PMC3176155

[47] Khristich AN, Mirkin SM. On the wrong DNA track: molecular mechanisms of repeat-mediated genome instability. J Biol Chem. 2020;295:4134–70. 10.1074/jbc.REV119.007678. 32060097 PMC7105313

[48] Peck LJ, Wang JC. Energetics of B-to-Z transition in DNA. Proc Natl Acad Sci USA. 1983;80:6206–10. 10.1073/pnas.80.20.6206. 6578505 PMC394264

[49] Rich A, Nordheim A, Wang AH. The chemistry and biology of left-handed Z-DNA. Annu Rev Biochem. 1984;53:791–846. 10.1146/annurev.bi.53.070184.004043. 6383204

[50] Petermann E, Lan L, Zou L. Sources, resolution and physiological relevance of R-loops and RNA–DNA hybrids. Nat Rev Mol Cell Biol. 2022;23:521–40. 10.1038/s41580-022-00474-x. 35459910

[51] Niehrs C, Luke B. Regulatory R-loops as facilitators of gene expression and genome stability. Nat Rev Mol Cell Biol. 2020;21:167–78. 10.1038/s41580-019-0206-3. 32005969 PMC7116639

[52] Cristini A, Tellier M, Constantinescu F et al. RNase H2, mutated in Aicardi-Goutières syndrome, resolves co-transcriptional R-loops to prevent DNA breaks and inflammation. Nat Commun. 2022;13:2961. 10.1038/s41467-022-30604-0. 35618715 PMC9135716

[53] Mirkin SM, Lyamichev VI, Drushlyak KN et al. DNA H form requires a homopurine-homopyrimidine mirror repeat. Nature. 1987;330:495–7. 10.1038/330495a0. 2825028

[54] Jain A, Wang G, Vasquez KM. DNA triple helices: biological consequences and therapeutic potential. Biochimie. 2008;90:1117–30. 10.1016/j.biochi.2008.02.011. 18331847 PMC2586808

[55] Kwok CK, Merrick CJ. G-quadruplexes: prediction, characterization, and biological application. Trends Biotechnol. 2017;35:997–1013. 10.1016/j.tibtech.2017.06.012. 28755976

[56] Wang G, Zhao J, Vasquez KM. Methods to determine DNA structural alterations and genetic instability. Methods. 2009;48:54–62. 10.1016/j.ymeth.2009.02.012. 19245837 PMC2693251

[57] Biffi G, Tannahill D, McCafferty J et al. Quantitative visualization of DNA G-quadruplex structures in human cells. Nat Chem. 2013;5:182–6. 10.1038/nchem.1548. 23422559 PMC3622242

[58] Agazie YM, Burkholder GD, Lee JS. Triplex DNA in the nucleus: direct binding of triplex-specific antibodies and their effect on transcription, replication and cell growth. Biochem J. 1996;316:461–6. 10.1042/bj3160461. 8687388 PMC1217372

[59] Lafer EM, Möller A, Nordheim A et al. Antibodies specific for left-handed Z-DNA. Proc Natl Acad Sci USA. 1981;78:3546–50. 10.1073/pnas.78.6.3546. 6943554 PMC319606

[60] Javadekar SM, Nilavar NM, Paranjape A et al. Characterization of G-quadruplex antibody reveals differential specificity for G4 DNA forms. DNA Res. 2020;27:dsaa024. 10.1093/dnares/dsaa024. 33084858 PMC7711166

[61] Johnson SA, Paul T, Sanford SL et al. BG4 antibody can recognize telomeric G-quadruplexes harboring destabilizing base modifications and lesions. Nucleic Acids Res. 2024;52:1763–78. 10.1093/nar/gkad1209. 38153143 PMC10939409

[62] Zeraati M, Langley DB, Schofield P et al. I-motif DNA structures are formed in the nuclei of human cells. Nat Chem. 2018;10:631–7. 10.1038/s41557-018-0046-3. 29686376

[63] Zanin I, Ruggiero E, Nicoletto G et al. Genome-wide mapping of i-motifs reveals their association with transcription regulation in live human cells. Nucleic Acids Res. 2023;51:8309–21. 10.1093/nar/gkad626. 37528048 PMC10484731

[64] Placido D, Brown BA 2nd, Lowenhaupt K et al. A left-handed RNA double helix bound by the Z alpha domain of the RNA-editing enzyme ADAR1. Structure. 2007;15:395–404. 10.1016/j.str.2007.03.001. 17437712 PMC2082211

[65] Ha SC, Kim D, Hwang HY et al. The crystal structure of the second Z-DNA binding domain of human DAI (ZBP1) in complex with Z-DNA reveals an unusual binding mode to Z-DNA. Proc Natl Acad Sci USA. 2008;105:20671–6. 10.1073/pnas.0810463106. 19095800 PMC2634953

[66] Chen L, Chen J-Y, Zhang X et al. R-ChIP using inactive RNase H reveals dynamic coupling of R-loops with transcriptional pausing at gene promoters. Mol Cell. 2017;68:745–57. 10.1016/j.molcel.2017.10.008. 29104020 PMC5957070

[67] Chen J-Y, Zhang X, Fu X-D et al. R-ChIP for genome-wide mapping of R-loops by using catalytically inactive RNASEH1. Nat Protoc. 2019;14:1661–85. 10.1038/s41596-019-0154-6. 30996261 PMC6604627

[68] Kouzine F, Gupta A, Baranello L et al. Transcription-dependent dynamic supercoiling is a short-range genomic force. Nat Struct Mol Biol. 2013;20:396–403. 10.1038/nsmb.2517. 23416947 PMC3594045

[69] Lahnsteiner A, Craig SJC, Kamali K et al. *In vivo* detection of DNA secondary structures using permanganate/S1 footprinting with direct adapter ligation and sequencing (PDAL-Seq). Methods Enzymol. 2024;695:159–91. 10.1016/bs.mie.2023.12.003. 38521584

[70] Wu T, Lyu R, You Q et al. Kethoxal-assisted single-stranded DNA sequencing captures global transcription dynamics and enhancer activity in situ. Nat Methods. 2020;17:515–23. 10.1038/s41592-020-0797-9. 32251394 PMC7205578

[71] Lyu R, Wu T, Zhu AC et al. KAS-seq: genome-wide sequencing of single-stranded DNA by N-kethoxal-assisted labeling. Nat Protoc. 2022;17:402–20. 10.1038/s41596-021-00647-6. 35013616 PMC8923001

[72] Chambers VS, Marsico G, Boutell JM et al. High-throughput sequencing of DNA G-quadruplex structures in the human genome. Nat Biotechnol. 2015;33:877–81. 10.1038/nbt.3295. 26192317

[73] Marsico G, Chambers VS, Sahakyan AB et al. Whole genome experimental maps of DNA G-quadruplexes in multiple species. Nucleic Acids Res. 2019;47:3862–74. 10.1093/nar/gkz179. 30892612 PMC6486626

[74] Kim N . The interplay between G-quadruplex and transcription. Curr Med Chem. 2019;26:2898–917. 10.2174/0929867325666171229132619. 29284393 PMC6026074

[75] Lafer EM, Sousa R, Rich A. Anti-Z-DNA antibody binding can stabilize Z-DNA in relaxed and linear plasmids under physiological conditions. EMBO J. 1985;4:3655–60. 10.1002/j.1460-2075.1985.tb04131.x. 4092691 PMC554714

[76] Boissieras J, Bonnet H, Susanto MF et al. iMab antibody binds single-stranded cytosine-rich sequences and unfolds DNA i-motifs. Nucleic Acids Res. 2024;52:8052–62. 10.1093/nar/gkae531. 38908025 PMC11317162

[77] Smolka JA, Sanz LA, Hartono SR et al. Recognition of RNA by the S9.6 antibody creates pervasive artifacts when imaging RNA:DNA hybrids. J. Cell Biol. 2021;220:e202004079. 10.1083/jcb.202004079. 33830170 PMC8040515

[78] Bou-Nader C, Bothra A, Garboczi DN et al. Structural basis of R-loop recognition by the S9.6 monoclonal antibody. Nat Commun. 2022;13:1641. 10.1038/s41467-022-29187-7. 35347133 PMC8960830

[79] Möller A, Gabriels JE, Lafer EM et al. Monoclonal antibodies recognize different parts of Z-DNA. J Biol Chem. 1982;257:12081–5. 10.1016/s0021-9258(18)33681-0. 7118931

[80] Run Y, Tavakoli M, Zhang Y et al. Formation and biological implications of Z-DNA. Trends Genet. 2025:42:163–76. 10.1016/j.tig.2025.07.006. 40803946 PMC12831796

[81] Martin RM, Almeida MR, Gameiro E et al. Live-cell imaging unveils distinct R-loop populations with heterogeneous dynamics. Nucleic Acids Res. 2023;51:11010–23. 10.1093/nar/gkad812. 37819055 PMC10639055

[82] Galli S, Melidis L, Flynn SM et al. DNA G-quadruplex recognition *in vitro* and in live cells by a structure-specific nanobody. J Am Chem Soc. 2022;144:23096–103. 10.1021/jacs.2c10656. 36488193 PMC9782783

[83] Di Antonio M, Ponjavic A, Radzevičius A et al. Single-molecule visualization of DNA G-quadruplex formation in live cells. Nat Chem. 2020;12:832–7. 10.1038/s41557-020-0506-4. 32690897 PMC7610488

[84] Summers PA, Lewis BW, Gonzalez-Garcia J et al. Visualising G-quadruplex DNA dynamics in live cells by fluorescence lifetime imaging microscopy. Nat Commun. 2021;12:162. 10.1038/s41467-020-20414-7. 33420085 PMC7794231

[85] Víšková P, Ištvánková E, Ryneš J et al. In-cell NMR suggests that DNA i-motif levels are strongly depleted in living human cells. Nat Commun. 2024;15:1992. 10.1038/s41467-024-46221-y. 38443388 PMC10914786

[86] Brázda V, Kolomazník J, Lýsek J et al. G4Hunter web application: a web server for G-quadruplex prediction. Bioinformatics. 2019;35:3493–5. 10.1093/bioinformatics/btz087. 30721922 PMC6748775

[87] Kikin O, D’Antonio L, Bagga PS. QGRS Mapper: a web-based server for predicting G-quadruplexes in nucleotide sequences. Nucleic Acids Res. 2006;34:W676–82. 10.1093/nar/gkl253. 16845096 PMC1538864

[88] Hon J, Martínek T, Zendulka J et al. pqsfinder: an exhaustive and imperfection-tolerant search tool for potential quadruplex-forming sequences in R. Bioinformatics. 2017;33:3373–9. 10.1093/bioinformatics/btx413. 29077807

[89] Garant J-M, Perreault J-P, Scott MS. Motif independent identification of potential RNA G-quadruplexes by G4RNA screener. Bioinformatics. 2017;33:3532–7. 10.1093/bioinformatics/btx498. 29036425 PMC5870565

[90] Sahakyan AB, Chambers VS, Marsico G et al. Machine learning model for sequence-driven DNA G-quadruplex formation. Sci Rep. 2017;7:14535. 10.1038/s41598-017-14017-4. 29109402 PMC5673958

[91] Cagirici HB, Budak H, Sen TZ. G4Boost: a machine learning-based tool for quadruplex identification and stability prediction. BMC Bioinformatics. 2022;23:240. 10.1186/s12859-022-04782-z. 35717172 PMC9206279

[92] Rocher V, Genais M, Nassereddine E et al. DeepG4: a deep learning approach to predict cell-type specific active G-quadruplex regions. PLoS Comput Biol. 2021;17:e1009308. 10.1371/journal.pcbi.1009308. 34383754 PMC8384162

[93] Korsakova A, Phan AT. Prediction of G4 formation in live cells with epigenetic data: a deep learning approach. NAR Genom Bioinform. 2023;5:lqad071. 10.1093/nargab/lqad071. 37636021 PMC10448861

[94] Hosseini M, Palmer A, Manka W et al. Deep statistical modelling of nanopore sequencing translocation times reveals latent non-B DNA structures. Bioinformatics. 2023;39:i242–51. 10.1093/bioinformatics/btad220. 37387144 PMC10311326

[95] Sawaya S, Boocock J, Black MA et al. Exploring possible DNA structures in real-time polymerase kinetics using pacific biosciences sequencer data. BMC Bioinformatics. 2015;16:21. 10.1186/s12859-014-0449-0. 25626999 PMC4384361

[96] Wang G, Mouratidis I, Provatas K et al. ZSeeker: an optimized algorithm for Z-DNA detection in genomic sequences. Brief Bioinform. 2025;26:bbaf240. 10.1093/bib/bbaf240. 40445004 PMC12123511

[97] Beknazarov N, Jin S, Poptsova M. Deep learning approach for predicting functional Z-DNA regions using omics data. Sci Rep. 2020;10:19134. 10.1038/s41598-020-76203-1. 33154517 PMC7644757

[98] Umerenkov D, Herbert A, Konovalov D et al. Z-flipon variants reveal the many roles of Z-DNA and Z-RNA in health and disease. Life Sci Alliance. 2023;6:e202301962. 10.26508/lsa.202301962. 37164635 PMC10172764

[99] Ho PS, Ellison MJ, Quigley GJ et al. A computer aided thermodynamic approach for predicting the formation of Z-DNA in naturally occurring sequences. EMBO J. 1986;5:2737–44. 10.1002/j.1460-2075.1986.tb04558.x. 3780676 PMC1167176

[100] Li K, Wu Z, Zhou J et al. R-loopAtlas: an integrated R-loop resource from 254 plant species sustained by a deep-learning-based tool. Mol Plant. 2023;16:493–6. 10.1016/j.molp.2022.12.012. 36536599

[101] Jenjaroenpun P, Wongsurawat T, Yenamandra SP et al. QmRLFS-finder: a model, web server and stand-alone tool for prediction and analysis of R-loop forming sequences. Nucleic Acids Res. 2015;43:W527–34. 10.1093/nar/gkv344. 25883153 PMC4489302

[102] Buske FA, Bauer DC, Mattick JS et al. Triplexator: detecting nucleic acid triple helices in genomic and transcriptomic data. Genome Res. 2012;22:1372–81. 10.1101/gr.130237.111. 22550012 PMC3396377

[103] Warwick T, Seredinski S, Krause NM et al. A universal model of RNA. DNA:DNA triplex formation accurately predicts genome-wide RNA–DNA interactions. Brief Bioinform. 2022;23:bbac445. 10.1093/bib/bbac445. 36239395 PMC9677506

[104] Zhang Y, Long Y, Kwoh CK. Deep learning based DNA:RNA triplex forming potential prediction. BMC Bioinformatics. 2020;21:522. 10.1186/s12859-020-03864-0. 33183242 PMC7663897

[105] Yu H, Li F, Yang B et al. iM-Seeker: a webserver for DNA i-motifs prediction and scoring via automated machine learning. Nucleic Acids Res. 2024;52:W19–28. 10.1093/nar/gkae315. 38676949 PMC11223794

[106] Yang B, Guneri D, Yu H et al. Prediction of DNA i-motifs via machine learning. Nucleic Acids Res. 2024;52:2188–97. 10.1093/nar/gkae092. 38364855 PMC10954440

[107] Yu H, Qi Y, Yang B et al. G4Atlas: a comprehensive transcriptome-wide G-quadruplex database. Nucleic Acids Res. 2023;51:D126–34. 10.1093/nar/gkac896. 36243987 PMC9825586

[108] Chantzi N, Nayak A, Baltoumas FA et al. Quadrupia provides a comprehensive catalog of G-quadruplexes across genomes from the tree of life. Genome Res. 2025;35:2578–600. 10.1101/gr.279790.124. 40858360 PMC12581993

[109] Qian SH, Shi M-W, Xiong Y-L et al. EndoQuad: a comprehensive genome-wide experimentally validated endogenous G-quadruplex database. Nucleic Acids Res. 2024;52:D72–80. 10.1093/nar/gkad966. 37904589 PMC10767823

[110] Dhapola P, Chowdhury S. QuadBase2: web server for multiplexed guanine quadruplex mining and visualization. Nucleic Acids Res. 2016;44:W277–83. 10.1093/nar/gkw425. 27185890 PMC4987949

[111] Provatas K, Chantzi N, Amptazi N et al. invertiaDB: a database of inverted repeats across organismal genomes. Nucleic Acids Res. 2025;53:gkaf329. 10.1093/nar/gkaf329. 40272360 PMC12019632

[112] Provatas K, Chantzi N, Patsakis M et al. Microsatellites explorer: a database of short tandem repeats across genomes. Comput Struct Biotechnol J. 2024;23:3817–26. 10.1016/j.csbj.2024.10.041. 39525087 PMC11550718

[113] Miller HE, Montemayor D, Li J et al. Exploration and analysis of R-loop mapping data with RLBase. Nucleic Acids Res. 2023;51:D1129–37. 10.1093/nar/gkac732. 36039757 PMC9825527

[114] Jenjaroenpun P, Wongsurawat T, Sutheeworapong S et al. R-loopDB: a database for R-loop forming sequences (RLFS) and R-loops. Nucleic Acids Res. 2017;45:D119–27. 10.1093/nar/gkw1054. 27899586 PMC5210542

[115] Cer RZ, Donohue DE, Mudunuri US et al. Non-B DB v2.0: a database of predicted non-B DNA-forming motifs and its associated tools. Nucleic Acids Res. 2013;41:D94–100. 10.1093/nar/gks955. 23125372 PMC3531222

[116] Prorok P, Artufel M, Aze A et al. Involvement of G-quadruplex regions in mammalian replication origin activity. Nat Commun. 2019;10:3274. 10.1038/s41467-019-11104-0. 31332171 PMC6646384

[117] Cayrou C, Coulombe P, Puy A et al. New insights into replication origin characteristics in metazoans. Cell Cycle. 2012;11:658. 10.4161/cc.11.4.19097. 22373526 PMC3318102

[118] Comoglio F, Schlumpf T, Schmid V et al. High-resolution profiling of drosophila replication start sites reveals a DNA shape and chromatin signature of metazoan origins. Cell Rep. 2015;11:821–34. 10.1016/j.celrep.2015.03.070. 25921534 PMC4562395

[119] Valton A-L, Hassan-Zadeh V, Lema I et al. G4 motifs affect origin positioning and efficiency in two vertebrate replicators. EMBO J. 2014;33:732–46. 10.1002/embj.201387506. 24521668 PMC4000090

[120] Takahashi S, Brazier JA, Sugimoto N. Topological impact of noncanonical DNA structures on Klenow fragment of DNA polymerase. Proc Natl Acad Sci USA. 2017;114:9605–10. 10.1073/pnas.1704258114. 28827350 PMC5594654

[121] Martinez P, David C, Zeraati M et al. Human genomic DNA is widely interspersed with i-motif structures. EMBO J. 2024;43:4786–804. 10.1038/s44318-024-00210-5. 39210146 PMC11480443

[122] Pearson CE, Zorbas H, Price GB et al. Inverted repeats, stem-loops, and cruciforms: significance for initiation of DNA replication. J Cell Biochem. 1996;63:1–22. 10.1002/(SICI)1097-4644(199610)63:1<1::AID-JCB1>3.0.CO;2-3. 8891900

[123] Wang S, Xu Y. Z-form DNA–RNA hybrid blocks DNA replication. Nucleic Acids Res. 2025;53:gkaf135. 10.1093/nar/gkaf135. 40037715 PMC11879439

[124] Wang G, Vasquez KM. Effects of replication and transcription on DNA structure-related genetic instability. Genes (Basel). 2017;8:17. 10.3390/genes8010017. 28067787 PMC5295012

[125] Huppert JL, Balasubramanian S. G-quadruplexes in promoters throughout the human genome. Nucleic Acids Res. 2007;35:406–13. 10.1093/nar/gkl1057. 17169996 PMC1802602

[126] Hänsel-Hertsch R, Beraldi D, Lensing SV et al. G-quadruplex structures mark human regulatory chromatin. Nat Genet. 2016;48:1267–72. 10.1038/ng.3662. 27618450

[127] Robinson J, Flint G, Garner I et al. G-quadruplex structures regulate long-range transcriptional reprogramming to promote drug resistance in ovarian cancer cells. Genome Biol. 2025;26:183. 10.1186/s13059-025-03654-y. 40646632 PMC12255116

[128] Sanz LA, Hartono SR, Lim YW et al. Prevalent, dynamic, and conserved R-loop structures associate with specific epigenomic signatures in mammals. Mol Cell. 2016;63:167–78. 10.1016/j.molcel.2016.05.032. 27373332 PMC4955522

[129] Gymrek M, Willems T, Guilmatre A et al. Abundant contribution of short tandem repeats to gene expression variation in humans. Nat Genet. 2016;48:22–9. 10.1038/ng.3461. 26642241 PMC4909355

[130] Wulfridge P, Yan Q, Rell N et al. G-quadruplexes associated with R-loops promote CTCF binding. Mol Cell. 2023;83:3064–79. 10.1016/j.molcel.2023.07.009. 37552993 PMC10529333

[131] Ginno PA, Lott PL, Christensen HC et al. R-loop formation is a distinctive characteristic of unmethylated human CpG island promoters. Mol Cell. 2012;45:814–25. 10.1016/j.molcel.2012.01.017. 22387027 PMC3319272

[132] Shin S-I, Ham S, Park J et al. Z-DNA-forming sites identified by ChIP-seq are associated with actively transcribed regions in the human genome. DNA Res. 2016;23:477–86. 10.1093/dnares/dsw031. 27374614 PMC5066173

[133] Lee K, Ku J, Ku D et al. Inverted Alu repeats: friends or foes in the human transcriptome. Exp Mol Med. 2024;56:1250–62. 10.1038/s12276-024-01177-3. 38871814 PMC11263572

[134] Bogard N, Linder J, Rosenberg AB et al. A deep neural network for predicting and engineering alternative polyadenylation. Cell. 2019;178:91–106. 10.1016/j.cell.2019.04.046. 31178116 PMC6599575

[135] Eddy J, Vallur AC, Varma S et al. G4 motifs correlate with promoter-proximal transcriptional pausing in human genes. Nucleic Acids Res. 2011;39:4975–83. 10.1093/nar/gkr079. 21371997 PMC3130262

[136] Fang Y, Bansal K, Mostafavi S et al. AIRE relies on Z-DNA to flag gene targets for thymic T cell tolerization. Nature. 2024;628:400–7. 10.1038/s41586-024-07169-7. 38480882 PMC11091860

[137] Yoo W, Song YW, Bansal V et al. G-quadruplex-dependent transcriptional regulation by molecular condensation in the Bcl3 promoter. Nucleic Acids Res. 2025;53:gkaf827. 10.1093/nar/gkaf827. 40884402 PMC12397910

[138] Beknazarov N, Konovalov D, Herbert A et al. Z-DNA formation in promoters conserved between human and mouse are associated with increased transcription reinitiation rates. Sci Rep. 2024;14:1–18. 10.1038/s41598-024-68439-y. 39090226 PMC11294368

[139] Lago S, Nadai M, Cernilogar FM et al. Promoter G-quadruplexes and transcription factors cooperate to shape the cell type-specific transcriptome. Nat Commun. 2021;12:3885. 10.1038/s41467-021-24198-2. 34162892 PMC8222265

[140] Li L, Williams P, Ren W et al. YY1 interacts with guanine quadruplexes to regulate DNA looping and gene expression. Nat Chem Biol. 2021;17:161–8. 10.1038/s41589-020-00695-1. 33199912 PMC7854983

[141] Brázda V, Coufal J. Recognition of local DNA structures by p53 protein. Int J Mol Sci. 2017;18:375. 10.3390/ijms18020375. 28208646 PMC5343910

[142] Cogoi S, Xodo LE. G-quadruplex formation within the promoter of the KRAS proto-oncogene and its effect on transcription. Nucleic Acids Res. 2006;34:2536–49. 10.1093/nar/gkl286. 16687659 PMC1459413

[143] Kaiser CE, Van Ert NA, Agrawal P et al. Insight into the complexity of the i-motif and G-quadruplex DNA structures formed in the KRAS promoter and subsequent drug-induced gene repression. J Am Chem Soc. 2017;139:8522–36. 10.1021/jacs.7b02046. 28570076 PMC5978000

[144] Kang H-J, Kendrick S, Hecht SM et al. The transcriptional complex between the BCL2 i-motif and hnRNP LL is a molecular switch for control of gene expression that can be modulated by small molecules. J Am Chem Soc. 2014;136:4172–85. 10.1021/ja4109352. 24559432 PMC3985447

[145] Roy B, Talukder P, Kang H-J et al. Interaction of individual structural domains of hnRNP LL with the BCL2 promoter i-motif DNA. J Am Chem Soc. 2016;138:10950–62. 10.1021/jacs.6b05036. 27483029

[146] Sutherland C, Cui Y, Mao H et al. A mechanosensor mechanism controls the G-quadruplex/i-motif molecular switch in the MYC promoter NHE III1. J Am Chem Soc. 2016;138:14138–51. 10.1021/jacs.6b09196. 27669098

[147] Qin G, Liu Z, Yang J et al. Targeting specific DNA G-quadruplexes with CRISPR-guided G-quadruplex-binding proteins and ligands. Nat Cell Biol. 2024;26:1212–24. 10.1038/s41556-024-01448-1. 38961283

[148] Esain-Garcia I, Kirchner A, Melidis L et al. G-quadruplex DNA structure is a positive regulator of transcription. Proc Natl Acad Sci USA. 2024;121:e2320240121. 10.1073/pnas.2320240121. 38315865 PMC10873556

[149] Doyle C, Herka K, Flynn SM et al. DNA G-quadruplex structures act as functional elements in α- and β-globin enhancers. Genome Biol. 2025;26:155. 10.1186/s13059-025-03627-1. 40468392 PMC12139101

[150] Ditlevson JV, Tornaletti S, Belotserkovskii BP et al. Inhibitory effect of a short Z-DNA forming sequence on transcription elongation by T7 RNA polymerase. Nucleic Acids Res. 2008;36:3163–70. 10.1093/nar/gkn136. 18400779 PMC2425487

[151] Belotserkovskii BP, De Silva E, Tornaletti S et al. A triplex-forming sequence from the human c-MYC promoter interferes with DNA transcription. J Biol Chem. 2007;282:32433–41. 10.1074/jbc.M704618200. 17785457

[152] Binas O, Tants J-N, Peter SA et al. Structural basis for the recognition of transiently structured AU-rich elements by Roquin. Nucleic Acids Res. 2020;48:7385–403. 10.1093/nar/gkaa465. 32491174 PMC7367199

[153] Matoulkova E, Michalova E, Vojtesek B et al. The role of the 3’ untranslated region in post-transcriptional regulation of protein expression in mammalian cells. RNA Biol. 2012;9:563–76. 10.4161/rna.20231. 22614827

[154] Hong D, Jeong S. 3’UTR diversity: expanding repertoire of RNA alterations in human mRNAs. Mol Cells. 2023;46:48–56. 10.14348/molcells.2023.0003. 36697237 PMC9880603

[155] Rothamel K, Arcos S, Kim B et al. ELAVL1 primarily couples mRNA stability with the 3’ UTRs of interferon-stimulated genes. Cell Rep. 2021;35:109178. 10.1016/j.celrep.2021.109178. 34038724 PMC8225249

[156] Lebedeva S, Jens M, Theil K et al. Transcriptome-wide analysis of regulatory interactions of the RNA-binding protein HuR. Mol Cell. 2011;43:340–52. 10.1016/j.molcel.2011.06.008. 21723171

[157] Bhattacharyya SN, Habermacher R, Martine U et al. Relief of microRNA-mediated translational repression in human cells subjected to stress. Cell. 2006;125:1111–24. 10.1016/j.cell.2006.04.031. 16777601

[158] Charlesworth A, Meijer HA, Moor CH. Specificity factors in cytoplasmic polyadenylation. Wiley Interdiscip Rev RNA. 2013;4:437–61. 10.1002/wrna.1171. 23776146 PMC3736149

[159] Brooks SA, Blackshear PJ. Tristetraprolin (TTP): interactions with mRNA and proteins, and current thoughts on mechanisms of action. Biochim Biophys Acta. 2013;1829:666–79. 10.1016/j.bbagrm.2013.02.003. 23428348 PMC3752887

[160] Siegel DA, Le Tonqueze O, Biton A et al. Massively parallel analysis of human 3’ UTRs reveals that AU-rich element length and registration predict mRNA destabilization. G3. 2022;12:jkab404. 10.1093/g3journal/jkab404. 34849835 PMC8728028

[161] Fabian MR, Frank F, Rouya C et al. Structural basis for the recruitment of the human CCR4-NOT deadenylase complex by tristetraprolin. Nat Struct Mol Biol. 2013;20:735–9. 10.1038/nsmb.2572. 23644599 PMC4811204

[162] Bulbrook D, Brazier H, Mahajan P et al. Tryptophan-mediated interactions between tristetraprolin and the CNOT9 subunit are required for CCR4-NOT deadenylase complex recruitment. J Mol Biol. 2018;430:722–36. 10.1016/j.jmb.2017.12.018. 29291391

[163] Carreño A, Lykke-Andersen J. The conserved CNOT1 interaction motif of Tristetraprolin regulates ARE-mRNA decay independently of the p38 MAPK-MK2 kinase pathway. Mol Cell Biol. 2022;42:e0005522. 10.1128/mcb.00055-22. 35920669 PMC9476947

[164] Mukherjee N, Jacobs NC, Hafner M et al. Global target mRNA specification and regulation by the RNA-binding protein ZFP36. Genome Biol. 2014;15:R12. 10.1186/gb-2014-15-1-r12. 24401661 PMC4053807

[165] Georgakopoulos-Soares I, Parada GE, Wong HY et al. Alternative splicing modulation by G-quadruplexes. Nat Commun. 2022;13:2404. 10.1038/s41467-022-30071-7. 35504902 PMC9065059

[166] Huang H, Zhang J, Harvey SE et al. RNA G-quadruplex secondary structure promotes alternative splicing via the RNA-binding protein hnRNPF. Genes Dev. 2017;31:2296. 10.1101/gad.305862.117. 29269483 PMC5769772

[167] Vo T, Brownmiller T, Hall K et al. HNRNPH1 destabilizes the G-quadruplex structures formed by G-rich RNA sequences that regulate the alternative splicing of an oncogenic fusion transcript. Nucleic Acids Res. 2022;50:6474–96. 10.1093/nar/gkac409. 35639772 PMC9226515

[168] Huertas P, Aguilera A. Cotranscriptionally formed DNA:RNA hybrids mediate transcription elongation impairment and transcription-associated recombination. Mol Cell. 2003;12:711–21. 10.1016/j.molcel.2003.08.010. 14527416

[169] Mischo HE, Gómez-González B, Grzechnik P et al. Yeast Sen1 helicase protects the genome from transcription-associated instability. Mol Cell. 2011;41:21–32. 10.1016/j.molcel.2010.12.007. 21211720 PMC3314950

[170] Yu K, Chedin F, Hsieh C-L et al. R-loops at immunoglobulin class switch regions in the chromosomes of stimulated B cells. Nat Immunol. 2003;4:442–51. 10.1038/ni919. 12679812

[171] Qiao Q, Wang L, Meng F-L et al. AID recognizes structured DNA for class switch recombination. Mol Cell. 2017;67:361–73. 10.1016/j.molcel.2017.06.034. 28757211 PMC5771415

[172] Larson ED, Duquette ML, Cummings WJ et al. MutSalpha binds to and promotes synapsis of transcriptionally activated immunoglobulin switch regions. Curr Biol. 2005;15:470–4. 10.1016/j.cub.2004.12.077. 15753043

[173] Chantzi N, Chan CSY, Patsakis M et al. Ribosomal DNA arrays are the most H-DNA rich element in the human genome. NAR Genom Bioinform. 2025;7:lqaf012. 10.1093/nargab/lqaf012. 40041207 PMC11879447

[174] Chantzi N, Liew SW, Wijaya A et al. Landscape and mutational dynamics of G-quadruplexes in the complete human genome and in haplotypes of diverse ancestry. bioRxiv, 10.1101/2025.06.17.660256, 25 June 2025, preprint: not peer reviewed.

[175] Wietmarschen N, Merzouk S, Halsema N et al. BLM helicase suppresses recombination at G-quadruplex motifs in transcribed genes. Nat Commun. 2018;9:271. 10.1038/s41467-017-02760-1. 29348659 PMC5773480

[176] Mani P, Yadav VK, Das SK et al. Genome-wide analyses of recombination prone regions predict role of DNA structural motif in recombination. PLoS One. 2009;4:e4399. 10.1371/journal.pone.0004399. 19198658 PMC2635932

[177] Rooney SM, Moore PD. Antiparallel, intramolecular triplex DNA stimulates homologous recombination in human cells. Proc Natl Acad Sci USA. 1995;92:2141–4. 10.1073/pnas.92.6.2141. 7892237 PMC42439

[178] Datta HJ, Chan PP, Vasquez KM et al. Triplex-induced recombination in human cell-free extracts. Dependence on XPA and HsRad51. J Biol Chem. 2001;276:18018–23. 10.1074/jbc.M011646200. 11278954

[179] Faruqi AF, Datta HJ, Carroll D et al. Triple-helix formation induces recombination in mammalian cells via a nucleotide excision repair-dependent pathway. Mol Cell Biol. 2000;20:990–1000. 10.1128/mcb.20.3.990-1000.2000. 10629056 PMC85216

[180] Tikhonova P, Pavlova I, Isaakova E et al. DNA G-quadruplexes contribute to CTCF recruitment. Int J Mol Sci. 2021;22:7090. 10.3390/ijms22137090. 34209337 PMC8269367

[181] Wulfridge P, Sarma K. Intertwining roles of R-loops and G-quadruplexes in DNA repair, transcription and genome organization. Nat Cell Biol. 2024;26:1025–36. 10.1038/s41556-024-01437-4. 38914786 PMC12044674

[182] Zhu M, Wang X, Zhao H et al. Update on R-loops in genomic integrity: formation, functions, and implications for human diseases. Genes Dis. 2025;12:101401. 10.1016/j.gendis.2024.101401. 40271193 PMC12017992

[183] Hou Y, Li F, Zhang R et al. Integrative characterization of G-quadruplexes in the three-dimensional chromatin structure. Epigenetics. 2019;14:894–911. 10.1080/15592294.2019.1621140. 31177910 PMC6691997

[184] Weintraub AS, Li CH, Zamudio AV et al. YY1 is a structural regulator of enhancer–promoter loops. Cell. 2017;171:1573–88. 10.1016/j.cell.2017.11.008. 29224777 PMC5785279

[185] Hegyi H . Enhancer–promoter interaction facilitated by transiently forming G-quadruplexes. Sci Rep. 2015;5:9165. 10.1038/srep09165. 25772493 PMC4360481

[186] DeMeis JD, Roberts JT, Delcher HA et al. Long G4-rich enhancers target promoters via a G4 DNA-based mechanism. Nucleic Acids Res. 2025;53:gkae1180. 10.1093/nar/gkae1180. 39658038 PMC11754661

[187] Mao S-Q, Ghanbarian AT, Spiegel J et al. DNA G-quadruplex structures mould the DNA methylome. Nat Struct Mol Biol. 2018;25:951. 10.1038/s41594-018-0131-8. 30275516 PMC6173298

[188] Temiz NA, Donohue DE, Bacolla A et al. The role of methylation in the intrinsic dynamics of B- and Z-DNA. PLoS One. 2012;7:e35558. 10.1371/journal.pone.0035558. 22530050 PMC3328458

[189] Crossley MP, Bocek M, Cimprich KA. R-loops as cellular regulators and genomic threats. Mol Cell. 2019;73:398–411. 10.1016/j.molcel.2019.01.024. 30735654 PMC6402819

[190] Meng Y, Wang G, He H et al. Z-DNA is remodelled by ZBTB43 in prospermatogonia to safeguard the germline genome and epigenome. Nat. Cell Biol. 2022;24:1141–53. 10.1038/s41556-022-00941-9. 35787683 PMC9276527

[191] Bestor T . Supercoiling-dependent sequence specificity of mammalian DNA methyltransferase. Nucleic Acids Res. 1987;15:3835–43. 10.1093/nar/15.9.3835. 3473446 PMC340785

[192] Esnault C, Zine E, Amal A et al. G-quadruplexes are promoter elements controlling nucleosome exclusion and RNA polymerase II pausing. Nat Genet. 2025;57:1981–93. 10.1038/s41588-025-02263-6. 40696173

[193] Vasquez KM, Wang G. The yin and yang of repair mechanisms in DNA structure-induced genetic instability. Mutat Res. 2013;743-744:118–31. 10.1016/j.mrfmmm.2012.11.005. 23219604 PMC3661696

[194] Casasnovas JM, Azorín F Supercoiled induced transition to the Z-DNA conformation affects the ability of a d(CG/GC)12 sequence to be organized into nucleosome-cores. Nucleic Acids Res. 1987;15:8899–918. 10.1093/nar/15.21.8899. 3684575 PMC306412

[195] Garner MM, Felsenfeld G. Effect of Z-DNA on nucleosome placement. J Mol Biol. 1987;196:581–90. 10.1016/0022-2836(87)90034-9. 3681969

[196] Bryan TM . G-quadruplexes at telomeres: friend or foe?. Molecules. 2020;25:3686. 10.3390/molecules25163686. 32823549 PMC7464828

[197] Sato K, Knipscheer P. G-quadruplex resolution: from molecular mechanisms to physiological relevance. DNA Repair (Amst.). 2023;130:103552. 10.1016/j.dnarep.2023.103552. 37572578

[198] Sakellariou D, Bak ST, Isik E et al. MutSβ regulates G4-associated telomeric R-loops to maintain telomere integrity in ALT cancer cells. Cell Rep. 2022;39:110602. 10.1016/j.celrep.2022.110602. 35385755

[199] Brown SL, Kendrick S. The i-motif as a molecular target: more than a complementary DNA secondary structure. Pharmaceuticals (Basel). 2021;14:96. 10.3390/ph14020096. 33513764 PMC7911047

[200] Smeds L, Kamali K, Kejnovská I et al. Non-canonical DNA in human and other ape telomere-to-telomere genomes. Nucleic Acids Res. 2025;53:gkaf298. 10.1093/nar/gkaf298. 40226919 PMC11995269

[201] Chantzi N, Georgakopoulos-Soares I. The repertoire of short tandem repeats across the tree of life. bioRxiv, 10.1101/2024.08.08.607201, 9 August 2024, preprint: not peer reviewed. PMC12699852 41388549

[202] Kasinathan S, Henikoff S. Non-B-form DNA is enriched at centromeres. Mol Biol Evol. 2018;35:949–62. 10.1093/molbev/msy010. 29365169 PMC5889037

[203] Mohanty SK, Chiaromonte F, Makova KD. Evolutionary dynamics of predicted G-quadruplexes in human and other great apes. Genome Biol. 2025;26:161. 10.1186/s13059-025-03635-1. 40500762 PMC12153182

[204] Liu C, Xu W, Wang L et al. Dual roles of R-loops in the formation and processing of programmed DNA double-strand breaks during meiosis. Cell Biosci. 2023;13:82. 10.1186/s13578-023-01026-2. 37170281 PMC10173651

[205] Yella VR, Vanaja A. Computational analysis on the dissemination of non-B DNA structural motifs in promoter regions of 1180 cellular genomes. Biochimie. 2023;214:101–11. 10.1016/j.biochi.2023.06.002. 37311475

[206] Chantzi N, Moeckel C, Chan CSY et al. Characterization of hairpin loops and cruciforms across 118,065 genomes spanning the tree of life. bioRxiv, 10.1101/2024.09.29.615628, 29 September 2024, preprint: not peer reviewed. PMC13155455 41934469

[207] Getz LJ, Brown JM, Sobot L et al. Attenuation of a DNA cruciform by a conserved regulator directs T3SS1 mediated virulence in *Vibrio parahaemolyticus*. Nucleic Acids Res. 2023;51:6156–71. 10.1093/nar/gkad370. 37158250 PMC10325908

[208] Kingsford CL, Ayanbule K, Salzberg SL. Rapid, accurate, computational discovery of Rho-independent transcription terminators illuminates their relationship to DNA uptake. Genome Biol. 2007;8:R22. 10.1186/gb-2007-8-2-r22. 17313685 PMC1852404

[209] Minero GAS, Møllebjerg A, Thiesen C et al. Extracellular G-quadruplexes and Z-DNA protect biofilms from DNase I, and G-quadruplexes form a DNAzyme with peroxidase activity. Nucleic Acids Res. 2024;52:1575–90. 10.1093/nar/gkae034. 38296834 PMC10939358

[210] Bohálová N, Cantara A, Bartas M et al. Analyses of viral genomes for G-quadruplex forming sequences reveal their correlation with the type of infection. Biochimie. 2021;186:13–27. 10.1016/j.biochi.2021.03.017. 33839192

[211] Qin G, Zhao C, Liu Y et al. RNA G-quadruplex formed in SARS-CoV-2 used for COVID-19 treatment in animal models. Cell Discov. 2022;8:86. 10.1038/s41421-022-00450-x. 36068208 PMC9447362

[212] Lan TCT, Allan MF, Malsick LE et al. Secondary structural ensembles of the SARS-CoV-2 RNA genome in infected cells. Nat Commun. 2022;13:1128. 10.1038/s41467-022-28603-2. 35236847 PMC8891300

[213] DeAntoneo C, Herbert A, Balachandran S. Z-form nucleic acid-binding protein 1 (ZBP1) as a sensor of viral and cellular Z-RNAs: walking the razor’s edge. Curr Opin Immunol. 2023;83:102347. 10.1016/j.coi.2023.102347. 37276820 PMC10526625

[214] Du X, Gertz EM, Wojtowicz D et al. Potential non-B DNA regions in the human genome are associated with higher rates of nucleotide mutation and expression variation. Nucleic Acids Res. 2014;42:12367–79. 10.1093/nar/gku921. 25336616 PMC4227770

[215] Guiblet WM, Cremona MA, Harris RS et al. Non-B DNA: a major contributor to small- and large-scale variation in nucleotide substitution frequencies across the genome. Nucleic Acids Res. 2021;49:1497–516. 10.1093/nar/gkaa1269. 33450015 PMC7897504

[216] Buisson R, Langenbucher A, Danae B et al. Passenger hotspot mutations in cancer driven by APOBEC3A and mesoscale genomic features. Science. 2019;364:eaaw2872. 10.1126/science.aaw2872. 31249028 PMC6731024

[217] Thakur J, Packiaraj J, Henikoff S. Sequence, chromatin and evolution of satellite DNA. Int J Mol Sci. 2021;22:4309. 10.3390/ijms22094309. 33919233 PMC8122249

[218] Achaz G, Coissac E, Netter P et al. Associations between inverted repeats and the structural evolution of bacterial genomes. Genetics. 2003;164:1279–89. 10.1093/genetics/164.4.1279. 12930739 PMC1462642

[219] Moxon R, Bayliss C, Hood D. Bacterial contingency loci: the role of simple sequence DNA repeats in bacterial adaptation. Annu Rev Genet. 2006;40:307–33. 10.1146/annurev.genet.40.110405.090442. 17094739

[220] Galen SC, Natarajan C, Moriyama H et al. Contribution of a mutational hot spot to hemoglobin adaptation in high-altitude Andean house wrens. Proc Natl Acad Sci USA. 2015;112:13958–63. 10.1073/pnas.1507300112. 26460028 PMC4653164

[221] Lavrov DV, Maikova OO, Pett W et al. Small inverted repeats drive mitochondrial genome evolution in Lake Baikal sponges. Gene. 2012;505:91–9. 10.1016/j.gene.2012.05.039. 22669046

[222] Tan S, Ma H, Wang J et al. DNA transposons mediate duplications via transposition-independent and -dependent mechanisms in metazoans. Nat Commun. 2021;12:4280. 10.1038/s41467-021-24585-9. 34257290 PMC8277862

[223] Yin Y, Fan H, Zhou B et al. Molecular mechanisms and topological consequences of drastic chromosomal rearrangements of muntjac deer. Nat Commun. 2021;12:6858. 10.1038/s41467-021-27091-0. 34824214 PMC8617201

[224] Chan YF, Marks ME, Jones FC et al. Adaptive evolution of pelvic reduction in sticklebacks by recurrent deletion of a Pitx1 enhancer. Science. 2010;327:302–5. 10.1126/science.1182213. 20007865 PMC3109066

[225] Smith SS . Evolutionary expansion of structurally complex DNA sequences. Cancer Genomics Proteomics. 2010;7:207–15. https://www.ncbi.nlm.nih.gov/pubmed/20656986. 20656986

[226] Lee DSM, Ghanem LR, Barash Y. Integrative analysis reveals RNA G-quadruplexes in UTRs are selectively constrained and enriched for functional associations. Nat Commun. 2020;11:527. 10.1038/s41467-020-14404-y. 31988292 PMC6985247

[227] Li G, Su G, Wang Y et al. Integrative genomic analyses of promoter G-quadruplexes reveal their selective constraint and association with gene activation. Commun Biol. 2023;6:625. 10.1038/s42003-023-05015-6. 37301913 PMC10257653

[228] Guiblet WM, DeGiorgio M, Cheng X et al. Selection and thermostability suggest G-quadruplexes are novel functional elements of the human genome. Genome Res. 2021;31:1136–49. 10.1101/gr.269589.120. 34187812 PMC8256861

[229] Kha DT, Wang G, Natrajan N et al. Pathways for double-strand break repair in genetically unstable Z-DNA-forming sequences. J Mol Biol. 2010;398:471–80. 10.1016/j.jmb.2010.03.035. 20347845 PMC2878134

[230] Biffi G, Tannahill D, Miller J et al. Elevated levels of G-quadruplex formation in human stomach and liver cancer tissues. PLoS One. 2014;9:e102711. 10.1371/journal.pone.0102711. 25033211 PMC4102534

[231] Bacolla A, Wells RD. Non-B DNA conformations as determinants of mutagenesis and human disease. Mol Carcinog. 2009;48:273–85. 10.1002/mc.20507. 19306308

[232] Mirkin SM . Expandable DNA repeats and human disease. Nature. 2007;447:932–40. 10.1038/nature05977. 17581576

[233] Hanna R, Flamier A, Barabino A et al. G-quadruplexes originating from evolutionary conserved L1 elements interfere with neuronal gene expression in Alzheimer’s disease. Nat Commun. 2021;12:1828. 10.1038/s41467-021-22129-9. 33758195 PMC7987966

[234] Anand RP, Shah KA, Niu H et al. Overcoming natural replication barriers: differential helicase requirements. Nucleic Acids Res. 2012;40:1091–105. 10.1093/nar/gkr836. 21984413 PMC3273818

[235] Lopes J, Piazza A, Bermejo R et al. G-quadruplex-induced instability during leading-strand replication. EMBO J. 2011;30:4033–46. 10.1038/emboj.2011.316. 21873979 PMC3209785

[236] Krasilnikova MM, Mirkin SM. Replication stalling at Friedreich’s ataxia (GAA)n repeats *in vivo*. Mol Cell Biol. 2004;24:2286–95. 10.1128/MCB.24.6.2286-2295.2004. 14993268 PMC355872

[237] Rider SD Jr, Gadgil RY, Hitch DC et al. Stable G-quadruplex DNA structures promote replication-dependent genome instability. J Biol Chem. 2022;298:101947. 10.1016/j.jbc.2022.101947. 35447109 PMC9142560

[238] Murat P, Guilbaud G, Sale JE. DNA polymerase stalling at structured DNA constrains the expansion of short tandem repeats. Genome Biol. 2020;21:209. 10.1186/s13059-020-02124-x. 32819438 PMC7441554

[239] Hamperl S, Cimprich KA. The contribution of co-transcriptional RNA:DNA hybrid structures to DNA damage and genome instability. DNA Repair (Amst). 2014;19:84–94. 10.1016/j.dnarep.2014.03.023. 24746923 PMC4051866

[240] Kaushal S, Freudenreich CH. The role of fork stalling and DNA structures in causing chromosome fragility. Genes Chromosomes Cancer. 2019;58:270–83. 10.1002/gcc.22721. 30536896 PMC7083089

[241] Lee WTC, Yin Y, Morten MJ et al. Single-molecule imaging reveals replication fork coupled formation of G-quadruplex structures hinders local replication stress signaling. Nat Commun. 2021;12:2525. 10.1038/s41467-021-22830-9. 33953191 PMC8099879

[242] Martella M, Pichiorri F, Chikhale RV et al. i-Motif formation and spontaneous deletions in human cells. Nucleic Acids Res. 2022;50:3445–55. 10.1093/nar/gkac158. 35253884 PMC8989526

[243] Tao S, Run Y, Monchaud D et al. i-Motif DNA: identification, formation, and cellular functions. Trends Genet. 2024;40:853–67. 10.1016/j.tig.2024.05.011. 38902139

[244] Castellano-Pozo M, García-Muse T, Aguilera A. R-loops cause replication impairment and genome instability during meiosis. EMBO Rep. 2012;13:923–9. 10.1038/embor.2012.119. 22878416 PMC3463965

[245] Gan W, Guan Z, Liu J et al. R-loop-mediated genomic instability is caused by impairment of replication fork progression. Genes Dev. 2011;25:2041–56. 10.1101/gad.17010011. 21979917 PMC3197203

[246] Belotserkovskii BP, Tornaletti S, D’Souza AD et al. R-loop generation during transcription: formation, processing and cellular outcomes. DNA Repair (Amst). 2018;71:69–81. 10.1016/j.dnarep.2018.08.009. 30190235 PMC6340742

[247] Mérida-Cerro JA, Maraver-Cárdenas P, Rondón AG et al. Rat1 promotes premature transcription termination at R-loops. Nucleic Acids Res. 2024;52:3623–35. 10.1093/nar/gkae033. 38281203 PMC11039981

[248] García-Muse T, Aguilera A. R loops: from physiological to pathological roles. Cell. 2019;179:604–18. 10.1016/j.cell.2019.08.055. 31607512

[249] Li TT, Vasquez KM. Multi-faceted roles of ERCC1-XPF nuclease in processing non-B DNA structures. DNA (Basel). 2022;2:231–47. 10.3390/dna2040017. 40766048 PMC12323751

[250] Wang G, Vasquez KM. Impact of alternative DNA structures on DNA damage, DNA repair, and genetic instability. DNA Repair (Amst). 2014;19:143–51. 10.1016/j.dnarep.2014.03.017. 24767258 PMC4216180

[251] Kamath-Loeb AS, Loeb LA, Johansson E et al. Interactions between the Werner syndrome helicase and DNA polymerase delta specifically facilitate copying of tetraplex and hairpin structures of the d(CGG)n trinucleotide repeat sequence. J Biol Chem. 2001;276:16439–46. 10.1074/jbc.M100253200. 11279038

[252] Paeschke K, Bochman ML, Garcia PD et al. Pif1 family helicases suppress genome instability at G-quadruplex motifs. Nature. 2013;497:458–62. 10.1038/nature12149. 23657261 PMC3680789

[253] Mendoza O, Bourdoncle A, Boulé J-B et al. G-quadruplexes and helicases. Nucleic Acids Res. 2016;44:1989–2006. 10.1093/nar/gkw079. 26883636 PMC4797304

[254] Dai Y-X, Guo H-L, Liu N-N et al. Structural mechanism underpinning *Thermus oshimai* Pif1-mediated G-quadruplex unfolding. EMBO Rep. 2022;23:e53874. 10.15252/embr.202153874. 35736675 PMC9253758

[255] Song Z-Y, Zhang X, Ai X et al. Structural mechanism of RECQ1 helicase in unfolding G-quadruplexes compared with duplex DNA. Nucleic Acids Res. 2025;53:gkaf877. 10.1093/nar/gkaf877. 40966504 PMC12445659

[256] Banco MT, Paul T, Jiang J et al. Structural basis for dual DNA and RNA specificity of the G-quadruplex-resolving DEAH-box helicase DHX36. Cell Rep. 2025;44:116136. 10.1016/j.celrep.2025.116136. 40833853 PMC12621225

[257] Batra SA, Benjamin KC et al. G-quadruplex-stalled eukaryotic replisome structure reveals helical inchworm DNA translocation. Science. 2025;387:eadt1978. 10.1126/science.adt1978. 40048517 PMC12338045

[258] Lansdorp P, Wietmarschen N. Helicases FANCJ, RTEL1 and BLM act on guanine quadruplex DNA *in vivo*. Genes (Basel). 2019;10:870. 10.3390/genes10110870. 31683575 PMC6896191

[259] Castillo BP, Segura-Bayona S, Koole W et al. FANCJ promotes DNA synthesis through G-quadruplex structures. EMBO J. 2014;33:2521–33. 10.15252/embj.201488663. 25193968 PMC4282361

[260] Lerner LK, Sale JE. Replication of G quadruplex DNA. Genes (Basel). 2019;10:95. 10.3390/genes10020095. 30700033 PMC6409989

[261] Wu CG, Spies M. G-quadruplex recognition and remodeling by the FANCJ helicase. Nucleic Acids Res. 2016;44:8742–53. 10.1093/nar/gkw574. 27342280 PMC5062972

[262] Paeschke K, Capra JA, Zakian VA. DNA replication through G-quadruplex motifs is promoted by the *Saccharomyces cerevisiae* Pif1 DNA helicase. Cell. 2011;145:678–91. 10.1016/j.cell.2011.04.015. 21620135 PMC3129610

[263] Wu Y, Brosh RMJ. G-quadruplex nucleic acids and human disease. FEBS J. 2010;277:3470–88. 10.1111/j.1742-4658.2010.07760.x. 20670277 PMC2923685

[264] Bhatia V, Barroso SI, García-Rubio ML et al. BRCA2 prevents R-loop accumulation and associates with TREX-2 mRNA export factor PCID2. Nature. 2014;511:362–5. 10.1038/nature13374. 24896180

[265] Sollier J, Stork CT, García-Rubio ML et al. Transcription-coupled nucleotide excision repair factors promote R-loop-induced genome instability. Mol Cell. 2014;56:777–85. 10.1016/j.molcel.2014.10.020. 25435140 PMC4272638

[266] Jalan M, Sharma A, Pei X et al. RAD52 resolves transcription-replication conflicts to mitigate R-loop induced genome instability. Nat Commun. 2024;15:7776. 10.1038/s41467-024-51784-x. 39237529 PMC11377823

[267] Hatchi E, Skourti-Stathaki K, Ventz S et al. BRCA1 recruitment to transcriptional pause sites is required for R-loop-driven DNA damage repair. Mol Cell. 2015;57:636–47. 10.1016/j.molcel.2015.01.011. 25699710 PMC4351672

[268] Ellis DA, Reyes-Martín F, Rodríguez-López M et al. R-loops and regulatory changes in chronologically ageing fission yeast cells drive non-random patterns of genome rearrangements. PLoS Genet. 2021;17:e1009784. 10.1371/journal.pgen.1009784. 34464389 PMC8437301

[269] De Magis A, Manzo SG, Russo M et al. DNA damage and genome instability by G-quadruplex ligands are mediated by R loops in human cancer cells. Proc Natl Acad Sci USA. 2019;116:816–25. 10.1073/pnas.1810409116. 30591567 PMC6338839

[270] Brickner JR, Garzon JL, Cimprich KA. Walking a tightrope: the complex balancing act of R-loops in genome stability. Mol Cell. 2022;82:2267–97. 10.1016/j.molcel.2022.04.014. 35508167 PMC9233011

[271] Helmrich A, Ballarino M, Tora L. Collisions between replication and transcription complexes cause common fragile site instability at the longest human genes. Mol Cell. 2011;44:966–77. 10.1016/j.molcel.2011.10.013. 22195969

[272] Xu H, Ye J, Zhang K-X et al. Chemoproteomic profiling unveils binding and functional diversity of endogenous proteins that interact with endogenous triplex DNA. Nat Chem. 2024;16:1811–21. 10.1038/s41557-024-01609-7. 39223307

[273] Jain A, Bacolla A, Del Mundo IM et al. DHX9 helicase is involved in preventing genomic instability induced by alternatively structured DNA in human cells. Nucleic Acids Res. 2013;41:10345–57. 10.1093/nar/gkt804. 24049074 PMC3905860

[274] Chakraborty P, Grosse F. Human DHX9 helicase preferentially unwinds RNA-containing displacement loops (R-loops) and G-quadruplexes. DNA Repair (Amst). 2011;10:654–65. 10.1016/j.dnarep.2011.04.013. 21561811

[275] Tang F, Wang Y, Gao Z et al. Polymerase η recruits DHX9 helicase to promote replication across guanine quadruplex structures. J Am Chem Soc. 2022;144:14016–20. 10.1021/jacs.2c05312. 35905379 PMC9378570

[276] Zhou H, Wu S, Li B et al. RNA G-quadruplex (rG4) exacerbates cellular senescence by mediating ribosome pausing. Protein Cell. 2025;16:953–67. 10.1093/procel/pwaf047. 40503845 PMC12698187

[277] Varshney D, Cuesta SM, Herdy B et al. RNA G-quadruplex structures control ribosomal protein production. Sci Rep. 2021;11:22735. 10.1038/s41598-021-01847-6. 34815422 PMC8611094

[278] Butt Y, Sakhtemani R, Mohamad-Ramshan R et al. Distinguishing preferences of human APOBEC3A and APOBEC3B for cytosines in hairpin loops, and reflection of these preferences in APOBEC-signature cancer genome mutations. Nat Commun. 2024;15:2369. 10.1038/s41467-024-46231-w. 38499553 PMC10948833

[279] Nakauma-González JA, Rijnders M, Noordsij MTW et al. Whole-genome mapping of APOBEC mutagenesis in metastatic urothelial carcinoma identifies driver hotspot mutations and a novel mutational signature. Cell Genom. 2024;4:100528. 10.1016/j.xgen.2024.100528. 38552621 PMC11019362

[280] McKinney JA, Wang G, Mukherjee A et al. Distinct DNA repair pathways cause genomic instability at alternative DNA structures. Nat Commun. 2020;11:236. 10.1038/s41467-019-13878-9. 31932649 PMC6957503

[281] Pavlova AV, Kubareva EA, Monakhova MV et al. Impact of G-quadruplexes on the regulation of genome integrity, DNA damage and repair. Biomolecules. 2021;11:1284. 10.3390/biom11091284. 34572497 PMC8472537

[282] Linke R, Limmer M, Juranek SA et al. The relevance of G-quadruplexes for DNA repair. Int J Mol Sci. 2021;22:12599. 10.3390/ijms222212599. 34830478 PMC8620898

[283] Zell J, Rota SF, Britton S et al. DNA folds threaten genetic stability and can be leveraged for chemotherapy. RSC Chem Biol. 2021;2:47–76. 10.1039/d0cb00151a. 35340894 PMC8885165

[284] Duardo RC, Guerra F, Pepe S et al. Non-B DNA structures as a booster of genome instability. Biochimie. 2023;214:176–92. 10.1016/j.biochi.2023.07.002. 37429410

[285] Liu G, Myers S, Chen X et al. Replication fork stalling and checkpoint activation by a PKD1 locus mirror repeat polypurine-polypyrimidine (Pu-Py) tract. J Biol Chem. 2012;287:33412–23. 10.1074/jbc.M112.402503. 22872635 PMC3460443

[286] Georgoulis A, Vorgias CE, Chrousos GP et al. Genome instability and γH2AX. Int J Mol Sci. 2017;18:1979. 10.3390/ijms18091979. 28914798 PMC5618628

[287] Huang D, Kraus WL. The expanding universe of PARP1-mediated molecular and therapeutic mechanisms. Mol Cell. 2022;82:2315–34. 10.1016/j.molcel.2022.02.021. 35271815 PMC9232969

[288] Scheibye-Knudsen M, Tseng A, Borch JM et al. Cockayne syndrome group A and B proteins converge on transcription-linked resolution of non-B DNA. Proc Natl Acad Sci USA. 2016;113:12502–7. 10.1073/pnas.1610198113. 27791127 PMC5098674

[289] Edwards AD, Marecki JC, Byrd AK et al. G-Quadruplex loops regulate PARP-1 enzymatic activation. Nucleic Acids Res. 2021;49:416–31. 10.1093/nar/gkaa1172. 33313902 PMC7797039

[290] Lonskaya I, Potaman VN, Shlyakhtenko LS et al. Regulation of poly(ADP-ribose) polymerase-1 by DNA structure-specific binding. J Biol Chem. 2005;280:17076–83. 10.1074/jbc.M413483200. 15737996

[291] Fan H, Chu J-Y. A brief review of short tandem repeat mutation. Genomics Proteomics Bioinformatics. 2007;5:7–14. 10.1016/S1672-0229(07)60009-6. 17572359 PMC5054066

[292] Bonneville R, Krook MA, Kautto EA et al. Landscape of microsatellite instability across 39 cancer types. JCO Precision Oncol. 2017;2017:PO.17.00073. 10.1200/PO.17.00073. PMC5972025 29850653

[293] Liu G, Chen X, Bissler JJ et al. Replication-dependent instability at (CTG) x (CAG) repeat hairpins in human cells. Nat Chem Biol. 2010;6:652–9. 10.1038/nchembio.416. 20676085 PMC2924473

[294] Xu P, Pan F, Roland C et al. Dynamics of strand slippage in DNA hairpins formed by CAG repeats: roles of sequence parity and trinucleotide interrupts. Nucleic Acids Res. 2020;48:2232–45. 10.1093/nar/gkaa036. 31974547 PMC7049705

[295] Loureiro JR, Castro AF, Figueiredo AS et al. Molecular mechanisms in pentanucleotide repeat diseases. Cells. 2022;11:205. 10.3390/cells11020205. 35053321 PMC8773600

[296] Yeetong P, Pongpanich M, Srichomthong C et al. TTTCA repeat insertions in an intron of YEATS2 in benign adult familial myoclonic epilepsy type 4. Brain. 2019;142:3360–6. 10.1093/brain/awz267. 31539032

[297] Malik I, Kelley CP, Wang ET et al. Molecular mechanisms underlying nucleotide repeat expansion disorders. Nat Rev Mol Cell Biol. 2021;22:589–607. 10.1038/s41580-021-00382-6. 34140671 PMC9612635

[298] Bacolla A, Ye Z, Ahmed Z et al. Cancer mutational burden is shaped by G4 DNA, replication stress and mitochondrial dysfunction. Prog Biophys Mol Biol. 2019;147:47–61. 10.1016/j.pbiomolbio.2019.03.004. 30880007 PMC6745008

[299] De S, Michor F. DNA secondary structures and epigenetic determinants of cancer genome evolution. Nat Struct Mol Biol. 2011;18:950–5. 10.1038/nsmb.2089. 21725294 PMC3963273

[300] Poetsch AR . The genomics of oxidative DNA damage, repair, and resulting mutagenesis. Comput Struct Biotechnol J. 2020;18:207–19. 10.1016/j.csbj.2019.12.013. 31993111 PMC6974700

[301] Clark DW, Phang T, Edwards MG et al. Promoter G-quadruplex sequences are targets for base oxidation and strand cleavage during hypoxia-induced transcription. Free Radic Biol Med. 2012;53:51–9. 10.1016/j.freeradbiomed.2012.04.024. 22583700 PMC3377816

[302] Zewail-Foote M, Del Mundo IMA, Klattenhoff AW et al. Oxidative damage within alternative DNA structures results in aberrant mutagenic processing. Nucleic Acids Res. 2025;53:gkaf066. 10.1093/nar/gkaf066. 39950343 PMC11826088

[303] Leach DR . Long DNA palindromes, cruciform structures, genetic instability and secondary structure repair. Bioessays. 1994;16:893–900. 10.1002/bies.950161207. 7840768

[304] Hagan CE, Warren GJ. Viability of palindromic DNA is restored by deletions occurring at low but variable frequency in plasmids of Escherichia coli. Gene. 1983;24:317–26. 10.1016/0378-1119(83)90092-6. 6357953

[305] Lewis SM . Palindromy is eliminated through a structure-specific recombination process in rodent cells. Nucleic Acids Res. 1999;27:2521–8. 10.1093/nar/27.12.2521. 10352181 PMC148456

[306] Lobachev KS, Shor BM, Tran HT et al. Factors affecting inverted repeat stimulation of recombination and deletion in *Saccharomyces cerevisiae*. Genetics. 1998;148:1507–24. 10.1093/genetics/148.4.1507. 9560370 PMC1460095

[307] Nasar F, Jankowski C, Nag DK. Long palindromic sequences induce double-strand breaks during meiosis in yeast. Mol Cell Biol. 2000;20:3449–58. 10.1128/MCB.20.10.3449-3458.2000. 10779335 PMC85638

[308] Kurahashi H, Emanuel BS. Long AT-rich palindromes and the constitutional t(11;22) breakpoint. Hum Mol Genet. 2001;10:2605–17. 10.1093/hmg/10.23.2605. 11726547

[309] Cunningham LA, Coté AG, Cam-Ozdemir C et al. Rapid, stabilizing palindrome rearrangements in somatic cells by the center-break mechanism. Mol Cell Biol. 2003;23:8740–50. 10.1128/MCB.23.23.8740-8750.2003. 14612414 PMC262683

[310] Losch FO, Bredenbeck A, Hollstein VM et al. Evidence for a large double-cruciform DNA structure on the X chromosome of human and chimpanzee. Hum Genet. 2007;122:337–43. 10.1007/s00439-007-0405-4. 17638018

[311] Zou X, Morganella S, Glodzik D et al. Short inverted repeats contribute to localized mutability in human somatic cells. Nucleic Acids Res. 2017;45:11213–21. 10.1093/nar/gkx731. 28977645 PMC5737083

[312] Shi M-J, Meng X-Y, Fontugne J et al. Identification of new driver and passenger mutations within APOBEC-induced hotspot mutations in bladder cancer. Genome Med. 2020;12:85. 10.1186/s13073-020-00781-y. 32988402 PMC7646471

[313] Fekairi S, Scaglione S, Chahwan C et al. Human SLX4 is a holliday junction resolvase subunit that binds multiple DNA repair/recombination endonucleases. Cell. 2009;138:78–89. 10.1016/j.cell.2009.06.029. 19596236 PMC2861413

[314] Miglietta G, Russo M, Capranico G. G-quadruplex–R-loop interactions and the mechanism of anticancer G-quadruplex binders. Nucleic Acids Res. 2020;48:11942–57. 10.1093/nar/gkaa944. 33137181 PMC7708042

[315] Rodriguez R, Miller KM, Forment JV et al. Small-molecule-induced DNA damage identifies alternative DNA structures in human genes. Nat Chem Biol. 2012;8:301–10. 10.1038/nchembio.780. 22306580 PMC3433707

[316] Sato K, Martin-Pintado N, Post H et al. Multistep mechanism of G-quadruplex resolution during DNA replication. Sci Adv. 2021;7:eabf8653. 10.1126/sciadv.abf8653. 34559566 PMC8462899

[317] Pennarun G, Granotier C, Hoffschir F et al. Role of ATM in the telomere response to the G-quadruplex ligand 360A. Nucleic Acids Res. 2008;36:1741–54. 10.1093/nar/gkn026. 18263609 PMC2275132

[318] Hasegawa D, Okabe S, Okamoto K et al. G-quadruplex ligand-induced DNA damage response coupled with telomere dysfunction and replication stress in glioma stem cells. Biochem Biophys Res Commun. 2016;471:75–81. 10.1016/j.bbrc.2016.01.176. 26845351 PMC5176359

[319] Alexandrov LB, Kim J, Haradhvala NJ et al. The repertoire of mutational signatures in human cancer. Nature. 2020;578:94–101. 10.1038/s41586-020-1943-3. 32025018 PMC7054213

[320] Piazza A, Adrian M, Samazan F et al. Short loop length and high thermal stability determine genomic instability induced by G-quadruplex-forming minisatellites. EMBO J. 2015;34:1718–34. 10.15252/embj.201490702. 25956747 PMC4475404

[321] Nik-Zainal S, Davies H, Staaf J et al. Landscape of somatic mutations in 560 breast cancer whole-genome sequences. Nature. 2016;534:47–54. 10.1038/nature17676. 27135926 PMC4910866

[322] Ellegren H . Microsatellites: simple sequences with complex evolution. Nat Rev Genet. 2004;5:435–45. 10.1038/nrg1348. 15153996

[323] Lynch M, Sung W, Morris K et al. A genome-wide view of the spectrum of spontaneous mutations in yeast. Proc Natl Acad Sci USA. 2008;105:9272–7. 10.1073/pnas.0803466105. 18583475 PMC2453693

[324] Matsuyama Z, Izumi Y, Kameyama M et al. The effect of CAT trinucleotide interruptions on the age at onset of spinocerebellar ataxia type 1 (SCA1). J Med Genet. 1999;36:546–8. https://www.ncbi.nlm.nih.gov/pubmed/10424816. 10424816 PMC1734401

[325] Cumming SA, Hamilton MJ, Robb Y et al. *De novo* repeat interruptions are associated with reduced somatic instability and mild or absent clinical features in myotonic dystrophy type 1. Eur J Hum Genet. 2018;26:1635–47. 10.1038/s41431-018-0156-9. 29967337 PMC6189127

[326] Ananda G, Hile SE, Breski A et al. Microsatellite interruptions stabilize primate genomes and exist as population-specific single nucleotide polymorphisms within individual human genomes. PLoS Genet. 2014;10:e1004498. 10.1371/journal.pgen.1004498. 25033203 PMC4102424

[327] Matuszek Z, Arbab M, Kesavan M et al. Base editing of trinucleotide repeats that cause Huntington’s disease and Friedreich's ataxia reduces somatic repeat expansions in patient cells and in mice. Nat Genet. 2025;57:1437–51. 10.1038/s41588-025-02172-8. 40419681 PMC12165863

[328] Richl T, Kuper J, Kisker C. G-quadruplex-mediated genomic instability drives SNVs in cancer. Nucleic Acids Res. 2024;52:2198–211. 10.1093/nar/gkae098. 38407356 PMC10954472

[329] Piazza A, Cui X, Adrian M et al. Non-canonical G-quadruplexes cause the hCEB1 minisatellite instability in *Saccharomyces cerevisiae*. eLife. 2017;6:e26884. 10.7554/eLife.26884. 28661396 PMC5491262

[330] Del Mundo IMA, Zewail-Foote M, Kerwin SM et al. Alternative DNA structure formation in the mutagenic human c-MYC promoter. Nucleic Acids Res. 2017;45:4929–43. 10.1093/nar/gkx100. 28334873 PMC5416782

[331] Largy E, Mergny J-L, Gabelica V. Role of alkali metal ions in G-quadruplex nucleic acid structure and stability. Met Ions Life Sci. 2016;16:203–58. 10.1007/978-3-319-21756-7_7. 26860303

[332] Kim BG, Evans HM, Dubins DN et al. Effects of salt on the stability of a G-quadruplex from the human c-MYC promoter. Biochemistry. 2015;54:3420–30. 10.1021/acs.biochem.5b00097. 25984914

[333] Del Mundo IMA, Vasquez KM, Wang G. Modulation of DNA structure formation using small molecules. Biochim Biophys Acta Mol Cell Res. 2019;1866:118539. 10.1016/j.bbamcr.2019.118539. 31491448 PMC6851491

[334] Papadopoulou C, Guilbaud G, Schiavone D et al. Nucleotide pool depletion induces g-quadruplex-dependent perturbation of gene expression. Cell Rep. 2015;13:2491–503. 10.1016/j.celrep.2015.11.039. 26686635 PMC4695339

[335] Diggins L, Ross D, Bhanot S et al. CD spectra reveal the state of G-quadruplexes and i-motifs in repeated and other DNA sequences. Biophys Rep (N Y). 2025;5:100187. 10.1016/j.bpr.2024.100187. 39608571 PMC11699388

[336] Kinniburgh AJ . A cis-acting transcription element of the c-myc gene can assume an H-DNA conformation. Nucleic Acids Res. 1989;17:7771–8. 10.1093/nar/17.19.7771. 2678005 PMC334884

[337] Wang W, Hu S, Gu Y et al. Human MYC G-quadruplex: from discovery to a cancer therapeutic target. Biochim Biophys Acta Rev Cancer. 2020;1874:188410. 10.1016/j.bbcan.2020.188410. 32827579

[338] Wölfl S, Wittig B, Rich A. Identification of transcriptionally induced Z-DNA segments in the human c-myc gene. Biochim Biophys Acta. 1995;1264:294–302. 10.1016/0167-4781(95)00155-7. 8547317

[339] Wittig B, Wölfl S, Dorbic T et al. Transcription of human c-myc in permeabilized nuclei is associated with formation of Z-DNA in three discrete regions of the gene. EMBO J. 1992;11:4653–63. 10.1002/j.1460-2075.1992.tb05567.x. 1330542 PMC557041

[340] Siddiqui-Jain A, Grand CL, Bearss DJ et al. Direct evidence for a G-quadruplex in a promoter region and its targeting with a small molecule to repress c-MYC transcription. Proc Natl Acad Sci USA. 2002;99:11593–8. 10.1073/pnas.182256799. 12195017 PMC129314

[341] Grand CL, Powell TJ, Nagle RB et al. Mutations in the G-quadruplex silencer element and their relationship to c-MYC overexpression, NM23 repression, and therapeutic rescue. Proc Natl Acad Sci USA. 2005;102:516. 10.1073/pnas.0408999101. PMC544325 15696627

[342] Li Z, Liu C, Huang C et al. Quinazoline derivative QPB-15e stabilizes the c-myc promoter G-quadruplex and inhibits tumor growth *in vivo*. Oncotarget. 2016;7:34266–76. 10.18632/oncotarget.9088. 27144522 PMC5085154

[343] Adachi M, Tsujimoto Y. Potential Z-DNA elements surround the breakpoints of chromosome translocation within the 5’ flanking region of bcl-2 gene. Oncogene. 1990;5:1653–7. https://www.ncbi.nlm.nih.gov/pubmed/2176281. 2176281

[344] Singh M, Gupta R, Comez L et al. BCL2 G quadruplex-binding small molecules: current status and prospects for the development of next-generation anticancer therapeutics. Drug Discov Today. 2022;27:2551–61. 10.1016/j.drudis.2022.06.002. 35709931

[345] Raghavan SC, Chastain P, Lee JS et al. Evidence for a triplex DNA conformation at the bcl-2 major breakpoint region of the t(14;18) translocation. J Biol Chem. 2005;280:22749–60. 10.1074/jbc.M502952200. 15840562

[346] Bielskutė S, Plavec J, Podbevšek P. Oxidative lesions modulate G-quadruplex stability and structure in the human BCL2 promoter. Nucleic Acids Res. 2021;49:2346–56. 10.1093/nar/gkab057. 33638996 PMC7913773

[347] Morgan RK, Batra H, Gaerig VC et al. Identification and characterization of a new G-quadruplex forming region within the kRAS promoter as a transcriptional regulator. Biochim Biophys Acta. 2016;1859:235–45. 10.1016/j.bbagrm.2015.11.004. 26597160

[348] Elliott K, Larsson E. Non-coding driver mutations in human cancer. Nat Rev Cancer. 2021;21:500–9. 10.1038/s41568-021-00371-z. 34230647

[349] Weinhold N, Jacobsen A, Schultz N et al. Genome-wide analysis of noncoding regulatory mutations in cancer. Nat Genet. 2014;46:1160–5. 10.1038/ng.3101. 25261935 PMC4217527

[350] Pavlova AV, Savitskaya VY, Dolinnaya NG et al. G-quadruplex formed by the promoter region of the hTERT gene: structure-driven effects on DNA mismatch repair functions. Biomedicines. 2022;10:1871. 10.3390/biomedicines10081871. 36009419 PMC9405553

[351] Song JH, Kang H-J, Luevano LA et al. Small-molecule-targeting hairpin loop of hTERT promoter G-quadruplex induces cancer cell death. Cell Chem Biol. 2019;26:1110–21.e4. 10.1016/j.chembiol.2019.04.009. 31155510 PMC6713458

[352] Seenisamy J, Rezler EM, Powell TJ et al. The dynamic character of the G-quadruplex element in the c-MYC promoter and modification by TMPyP4. J Am Chem Soc. 2004;126:8702–9. 10.1021/ja040022b. 15250722

[353] Balasubramanian S, Hurley LH, Neidle S. Targeting G-quadruplexes in gene promoters: a novel anticancer strategy?. Nat Rev Drug Discov. 2011;10:261–75. 10.1038/nrd3428. 21455236 PMC3119469

[354] Hussein Y . G-quadruplex DNA: a potential target for anti-cancer drug design. 2003. 10.1016/s0165-6147(00)01457-7 10740289

[355] Erwin GS, Gürsoy G, Al-Abri R et al. Recurrent repeat expansions in human cancer genomes. Nature. 2023;613:96–102. 10.1038/s41586-022-05515-1. 36517591 PMC9812771

[356] Stratton MR, Campbell PJ, Futreal PA. The cancer genome. Nature. 2009;458:719–24. 10.1038/nature07943. 19360079 PMC2821689

[357] Wong JKL, Aichmüller C, Schulze M et al. Association of mutation signature effectuating processes with mutation hotspots in driver genes and non-coding regions. Nat Commun. 2022;13:178. 10.1038/s41467-021-27792-6. 35013316 PMC8748499

[358] Fredriksson NJ, Ny L, Nilsson JA et al. Systematic analysis of noncoding somatic mutations and gene expression alterations across 14 tumor types. Nat Genet. 2014;46:1258–63. 10.1038/ng.3141. 25383969

[359] Johnson JE, Cao K, Ryvkin P et al. Altered gene expression in the Werner and Bloom syndromes is associated with sequences having G-quadruplex forming potential. Nucleic Acids Res. 2010;38:1114–22. 10.1093/nar/gkp1103. 19966276 PMC2831322

[360] Bacolla A, Wang G, Jain A et al. Non-B DNA-forming sequences and WRN deficiency independently increase the frequency of base substitution in human cells. J Biol Chem. 2011;286:10017–26. 10.1074/jbc.M110.176636. 21285356 PMC3060453

[361] Bharathi VP, Garruto RM et al. Role of DNA dynamics in Alzheimer’s disease. Brain Res Rev. 2008;58:136–48. 10.1016/j.brainresrev.2008.01.001. 18342372

[362] Suram A, Rao KSJ, Latha KS et al. First evidence to show the topological change of DNA from B-dNA to Z-DNA conformation in the hippocampus of Alzheimer’s brain. Neuromolecular Med. 2002;2:289–97. 10.1385/nmm:2:3:289. 12622407

[363] Handsaker RE, Kashin S, Reed NM et al. Long somatic DNA-repeat expansion drives neurodegeneration in Huntington’s disease. Cell. 2025;188:623–39. 10.1016/j.cell.2024.11.038. 39824182 PMC11822645

[364] Haeusler AR, Donnelly CJ, Periz G et al. C9orf72 nucleotide repeat structures initiate molecular cascades of disease. Nature. 2014;507:195–200. 10.1038/nature13124. 24598541 PMC4046618

[365] Mandke P, Kompella P, Wang G et al. Aging increases short, inverted repeat-mediated genomic instability *in vivo*. Aging Cell. 2025:24:e70250. 10.1111/acel.70250. 41025428 PMC12686595

[366] Jauregui-Lozano J, Escobedo S, Easton A et al. Proper control of R-loop homeostasis is required for maintenance of gene expression and neuronal function during aging. Aging Cell. 2022;21:e13554. 10.1111/acel.13554. 35048512 PMC8844117

[367] Damas J, Carneiro J, Gonçalves J et al. Mitochondrial DNA deletions are associated with non-B DNA conformations. Nucleic Acids Res. 2012;40:7606–21. 10.1093/nar/gks500. 22661583 PMC3439893

[368] Butler TJ, Estep KN, Sommers JA et al. Mitochondrial genetic variation is enriched in G-quadruplex regions that stall DNA synthesis *in vitro*. Hum Mol Genet. 2020;29:1292–309. 10.1093/hmg/ddaa043. 32191790 PMC7254849

[369] Dong DW, Pereira F, Barrett SP et al. Association of G-quadruplex forming sequences with human mtDNA deletion breakpoints. BMC Genomics [Electronic Resource]. 2014;15:677. 10.1186/1471-2164-15-677. 25124333 PMC4153896

[370] Simpson JT, Workman RE, Zuzarte PC et al. Detecting DNA cytosine methylation using nanopore sequencing. Nat Methods. 2017;14:407–10. 10.1038/nmeth.4184. 28218898

[371] Rand AC, Jain M, Eizenga JM et al. Mapping DNA methylation with high-throughput nanopore sequencing. Nat Methods. 2017;14:411–3. 10.1038/nmeth.4189. 28218897 PMC5704956

[372] Clark TA, Lu X, Luong K et al. Enhanced 5-methylcytosine detection in single-molecule, real-time sequencing via Tet1 oxidation. BMC Biol. 2013;11:4. 10.1186/1741-7007-11-4. 23339471 PMC3598637

[373] Liao W-W, Asri M, Ebler J et al. A draft human pangenome reference. Nature. 2023;617:312–24. 10.1038/s41586-023-05896-x. 37165242 PMC10172123

[374] Gao Y, Yang X, Chen H et al. A pangenome reference of 36 Chinese populations. Nature. 2023;619:112–21. 10.1038/s41586-023-06173-7. 37316654 PMC10322713

[375] Nurk S, Koren S, Rhie A et al. The complete sequence of a human genome. Science. 2022:376:44–53. 10.1126/science.abj6987. 35357919 PMC9186530

[376] Tsukakoshi K, Saito S, Yoshida W et al. CpG methylation changes G-quadruplex structures derived from gene promoters and interaction with VEGF and SP1. Molecules. 2018;23:944. 10.3390/molecules23040944. 29670067 PMC6017926

[377] Zyner KG, Simeone A, Flynn SM et al. G-quadruplex DNA structures in human stem cells and differentiation. Nat Commun. 2022;13:142. 10.1038/s41467-021-27719-1. 35013231 PMC8748810

[378] Niu K, Xiang L, Zhang X et al. DNA 5mC methylation inhibits the formation of G-quadruplex structures in the genome. Genome Biol. 2025;26:202. 10.1186/s13059-025-03678-4. 40646558 PMC12255034

[379] Zaichuk T, Marko JF. Single-molecule micromanipulation studies of methylated DNA. Biophys J. 2021;120:2148–55. 10.1016/j.bpj.2021.03.039. 33838135 PMC8390797

[380] Guiblet WM, Cremona MA, Cechova M et al. Long-read sequencing technology indicates genome-wide effects of non-B DNA on polymerization speed and error rate. Genome Res. 2018;28:1767–78. 10.1101/gr.241257.118. 30401733 PMC6280752

[381] McGinty RJ, Sunyaev SR. Revisiting mutagenesis at non-B DNA motifs in the human genome. Nat Struct Mol Biol. 2023;30:417–24. 10.1038/s41594-023-00936-6. 36914796 PMC10225297

[382] Taylor DJ, Eizenga JM, Li Q et al. Beyond the human genome project: the age of complete human genome sequences and pangenome references. Annu Rev Genomics Hum Genet. 2024;25:77–104. 10.1146/annurev-genom-021623-081639. 38663087 PMC11451085

[383] Rzhetsky A, Shatkay H, Wilbur WJ. How to get the most out of your curation effort. PLoS Comput Biol. 2009;5:e1000391. 10.1371/journal.pcbi.1000391. 19461884 PMC2678295

[384] Stein M, Hile SE, Weissensteiner MH et al. Variation in G-quadruplex sequence and topology differentially impacts human DNA polymerase fidelity. DNA Repair (Amst). 2022;119:103402. 10.1016/j.dnarep.2022.103402. 36116264 PMC9798401

[385] Martincorena I, Roshan A, Gerstung M et al. Tumor evolution. High burden and pervasive positive selection of somatic mutations in normal human skin. Science. 2015;348:880–6. 10.1126/science.aaa6806. 25999502 PMC4471149

[386] Martincorena I, Fowler JC, Wabik A et al. Somatic mutant clones colonize the human esophagus with age. Science. 2018;362:911. 10.1126/science.aau3879. 30337457 PMC6298579

[387] Abascal F, Harvey LMR, Mitchell E et al. Somatic mutation landscapes at single-molecule resolution. Nature. 2021;593:405–10. 10.1038/s41586-021-03477-4. 33911282

[388] Choudhury S, Huang AY, Kim J et al. Somatic mutations in single human cardiomyocytes reveal age-associated DNA damage and widespread oxidative genotoxicity. Nat Aging. 2022;2:714–25. 10.1038/s43587-022-00261-5. 36051457 PMC9432807

[389] Serrano IM, Hirose M, Valentine CC et al. Mitochondrial somatic mutation and selection throughout ageing. Nat Ecol Evol. 2024;8:1021–34. 10.1038/s41559-024-02338-3. 38361161 PMC11090800

[390] Hui WWI, Simeone A, Zyner KG et al. Single-cell mapping of DNA G-quadruplex structures in human cancer cells. Sci Rep. 2021;11:23641. 10.1038/s41598-021-02943-3. 34880271 PMC8654944

